# Oxylipins at intermediate larval stages of damselfly *Coenagrion hastulatum* as biochemical biomarkers for anthropogenic pollution

**DOI:** 10.1007/s11356-021-12503-x

**Published:** 2021-01-29

**Authors:** Jana Späth, Tomas Brodin, Daniel Cerveny, Richard Lindberg, Jerker Fick, Malin L. Nording

**Affiliations:** 1 Department of Chemistry, Umeå University, 90187 Umeå, SE Sweden; 2 Department of Ecology and Environmental Science, Umeå University, 90187 Umea, SE Sweden; 3 Department of Wildlife, Fish, and Environmental Studies, Swedish University of Agricultural Sciences, 90183 Umea, SE Sweden; 4 University of South Bohemia in Ceske Budejovice, Faculty of Fisheries and Protection of Waters, South Bohemian Research Center of Aquaculture and Biodiversity of Hydrocenoses, Zatisi 728/II, Vodnany, Czech Republic

**Keywords:** Benthic invertebrate, Life cycle, Liquid chromatography-mass spectrometry, Fatty acid metabolite, Eicosanoid, Biomarker, Variation

## Abstract

Aquatic pollution resulting from anthropogenic activities requires adequate environmental monitoring strategies in sentinel organisms. Thus, biochemical biomarkers have been used as early-warning tools of biological effects in aquatic organisms. However, before using these markers for environmental monitoring, knowledge about their developmental variation is vital. In this study, we assessed baseline levels and developmental variations of a group of potential biomarkers, oxylipins, during the lifespan of the Northern damselfly (*Coenagrion hastulatum*) using liquid chromatography-tandem mass spectrometry. Effects of wastewater exposure on baseline levels were studied in a subset of damselflies to investigate the responsiveness due to anthropogenic pollution. Thirty-eight oxylipins deriving from four polyunsaturated fatty acids via two enzymatic pathways were detected in damselflies at three larval stages and in the adult form. Overall, oxylipin baseline levels showed developmental variation, which was lowest in the intermediate larval stages. Effects of exposure to wastewater effluent on oxylipin baseline levels were dependent on the life stage and were greatest in the early and intermediate larval stages. The study provides first insights into oxylipin profiles of damselflies at different stages of development and their developmental variation. Based on our results, we propose further strategies for incorporating oxylipins in damselfly larvae as biochemical markers for anthropogenic pollution.

**Supplementary Information:**

The online version contains supplementary material available at 10.1007/s11356-021-12503-x.

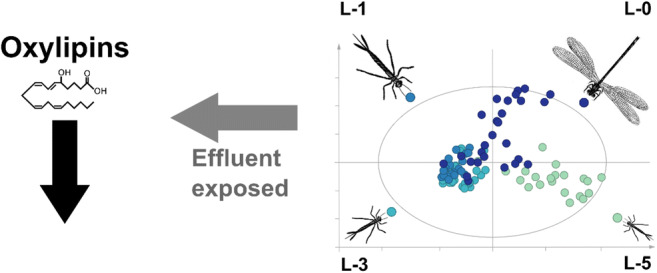

## Introduction

Globally, levels of environmental pollutants have increased in the aquatic environment due to anthropogenic activities (Halling-Sørensen et al. 1998; Luo et al. 2014; Häder et al. 2020). These pollutants can have adverse effects on individual aquatic organisms or even entire ecosystems (Daughton and Ternes 1999; Fleeger et al. 2003; Kidd et al. 2007). To assess the environmental risks associated with exposure to environmental pollutants and help to avoid long-term and irreversible effects, reliable monitoring systems are required.

Rather than measuring the pollutant load of aquatic ecosystems, biomarkers in a sentinel organism are increasingly being used as early warning tools in environmental monitoring (Depledge and Fossi 1994). Biological responses to a chemical or chemicals that give a measure of exposure and sometimes also toxic effects are utilized. Biochemical biomarkers are defined as substances that can be measured in body fluids, tissues or whole organisms and have the potential to be altered by exposure to pollutants and other environmental stressors (Depledge and Fossi 1994; van der Oost et al. 2003; Monserrat et al. 2007; Storhaug et al. 2019; Previšić et al. 2020).

A range of biochemical biomarkers has been studied in different aquatic organisms to evaluate their potential to reflect contamination in aquatic environments. Among these, several enzymes, e.g., catalase (CAT), cholinesterase (ChE), glutathione S-transferase (GST), and cytochrome P450 (CYP 450), are some of the most utilized biochemical markers of chemical pollution (Sarkar et al. 2006; Wigh et al. 2017; Rodrigues et al. 2019). Oxylipins represent a new group of putative biochemical biomarkers. They are potent fatty acid metabolites formed by oxidation of polyunsaturated fatty acids via three enzymatic pathways, i.e., the cyclooxygenase (COX), lipoxygenase (LOX), and CYP 450 pathways (Yang et al. 2009), and include the eicosanoids derived from arachidonic acid (AA). Oxylipins are promising biochemical markers as they may reflect alteration of several enzymatic pathways simultaneously, and thus better indicate the response of an organism to pollution by a complex mixture of otherwise extraneous substances. They regulate numerous metabolic functions, such as reproduction, inflammation, and immune cell behavior, in both vertebrates and invertebrates (Heckmann et al. 2008; Dennis and Norris 2015). Oxylipins have been studied in other invertebrate species such as the crustacean *Daphnia magna* (Heckmann et al. 2008; Garreta-Lara et al. 2018) and the barnacle *Balanus amphitrite* (Knight et al. 1999). In fish, levels of a group of oxylipins (prostaglandins) were altered after exposure to wastewater treatment plant effluent (David et al. 2017). In a previous pilot study, we found evidence of altered levels of oxylipins in response to exposure to wastewater treatment plant effluents (Späth et al. 2020). However, further research is needed to determine whether oxylipins may be useful biomarkers in environmental monitoring.

Prior to using specific biochemical markers for environmental monitoring, knowledge about their baseline levels in a sentinel organism is vital to differentiate between natural variation and pollutant-induced stress (van der Oost et al. 2003). Natural variation can be affected by a plethora of factors, including the individual’s developmental stage (Depledge and Fossi 1994). In the present study, baseline levels and developmental variation of oxylipins were studied during the lifespan of an invertebrate species, namely damselfly larvae.

Damselfly larvae were chosen as sentinel organism due to their ecological importance and wide distribution (Jonsson et al. 2014). Furthermore, damselfly larvae have been used to monitor effects of exposure to a variety of pollutants (Boroń and Mirosławski 2009; Janssens and Stoks 2013; Jonsson et al. 2014). However, oxylipins in damselfly larvae have only been studied to a limited extent.

The main objective of this study was to identify biomarker candidates among the investigated oxylipins for which the responsiveness to environmental pollution exceeded the developmental variation, detectable with an appropriate sample size. Hence, we investigated the developmental variability of oxylipins due to different maternal origin and across three larval stages, as well as in the adult stage of the northern damselfly (*Coenagrion hastulatum;* [*C.H.*]), and compared it to the effect of exposure to wastewater effluent, representing a real complex mixture of anthropogenic pollutants. We hypothesized that developmental variability would have an impact on the capacity of oxylipins to indicate exposure. Developmental variability in various invertebrate species has been established for biochemical biomarkers such as CAT, ChE, and GST (Scarduelli et al. 2017) but remains unknown for oxylipins. However, it is likely that also the fatty acid metabolism depends on developmental factors and has an impact on the levels and variability of oxylipins. This study is unique in its design as it provides first insights into baseline levels and developmental variation of oxylipin profiles in damselflies at different life stages. Based on our results, we propose further strategies for incorporating oxylipins in damselfly larvae as biomarkers that may be useful in future biomonitoring studies.

## Materials and methods

### Rearing conditions

**Fig. 1:**
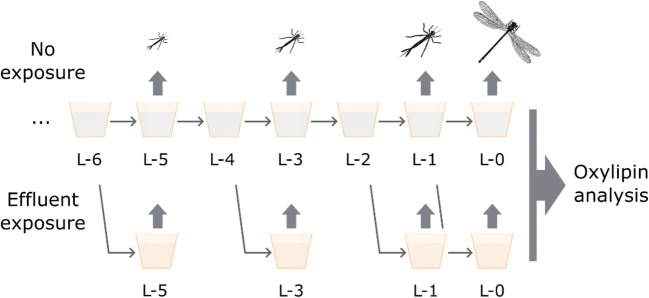
Study design. Damselfly larvae were reared individually until they reached their preselected stage (L-5, L-3, L-1, or L-0) at which the oxylipin analysis was carried out. A subset of larvae was exposed to wastewater effluent for the duration of one stage prior to the stage selected for oxylipin analysis (L-6 until L-5, L-4 until L-3, L-2 until L-1, or L-1 until L-0)

Four females, in copula, of the northern damselfly *C.H.* were collected in July 2018 at Lake Nydalasjön in Umeå, Sweden and brought to an aquatic laboratory at Umeå University for egg laying in wet filter paper. The filter papers were then submerged in an aerated tank filled with aged tap water until the eggs hatched. The hatchlings (*N* = 114) were placed individually in small aquaria (10 × 10 cm) containing non-chlorine aerated tap water and throughout their lives kept under standard conditions of light (14:10 L:D) and temperature (20 °C). The maternal origin was tracked for all larvae. The larvae were fed three times per week with size-matched zooplankton obtained from a local pond as long as weather conditions allowed, then daily with brine shrimp (*Artemia salina*) and zooplankton cultivated in-house. The larvae were checked daily for survival and change of developmental stage, i.e., instar, indicated by exoskeleton shedding. From hatching to emergence, damselfly *C.H.* undergoes in total eleven instars (Norling 1984). For randomization purposes, the instar at which sampling for oxylipin analysis took place was preselected for each individual. In total, three instars (L-5, L-3, L-1) were selected together with the final stage (L-0), where the larva emerged to the adult (Fig. 1). When the larvae had molted into the preselected instar, they were placed individually in 2-mL microcentrifuge tubes and stored at − 20 °C until analysis (*n* = 21, 24, 21, 27 for L-5, L-3, L-1, L-0, respectively). For a pilot study on exposure effects, the aerated water of a subset of larvae (*N* = 21) was replaced by wastewater effluent (obtained from a local wastewater treatment plant and stored at − 20 °C) for the duration of one larval stage prior to the preselected stage, e.g., larvae selected for L-1 were exposed from the time they reached L-2 until they molted into L-1 (see Fig. 1; *n* = 6, 6, 3, 6 for L-5, L-3, L-1, and L-0, respectively). On average, this resulted in exposure duration of 12, 22, 16, and 34 days for L-5, L-3, L-1, and L-0, respectively. Hence, there were no exposures throughout life included in the study design. The local wastewater treatment plant applies mechanical, chemical (flocculation with FeCl_3_), and biological (active sludge) treatment techniques, while no treatments for removal of nitrogen or disinfecting the wastewater are used (see Supplementary information for further information). The lab breeding was carried out between July 2018 and February 2019.

### Extraction

Extractions were performed from whole-body individuals (0.7–7 mg wet weight; see Supplementary information for wet weight and exoskeleton length of individual damselflies). 1.5 mL acetonitrile/water (90/10), containing nine internal standards (0.33 ng/mL of 12,13-DiHOME-d4 and 20-HETE-d6, 0.67 ng/mL of 12(13)-EpOME-d4, and 0.17 ng/mL of 9-HODE-d4, PGE2-d4, TXB2-d4, PGD2-d4, 5-HETE-d8, and 11(12)-EpETrE-d11, respectively) were added to the larvae. Homogenization was performed using stainless steel beads (3-mm diameter) added to each sample with shaking for 3 min at 30 Hz in a mixer mill (MM400, Retsch Technology, Haan, Germany). After centrifugation for 10 min at 14000 RPM, the supernatant was withdrawn, and samples were re-extracted with 1.5 mL 90/10 acetonitrile/water following the above described procedure. Finally, supernatants were combined and a 1.5 mL aliquot was transferred to falcon tubes and evaporated under vacuum (MiniVac system, Farmingdale, NY, USA). Residues were reconstituted in 110 μL methanol containing the recovery standard (0.5 ng/mL 12-[[(cyclohexylamino)carbonyl]amino]-dodecanoic acid [CUDA]) and transferred to vials for further analysis. All internal standards and CUDA were purchased from Cayman Chemical (Ann Arbor, MI, USA). Acetonitrile, isopropanol, and methanol were from Merck (Darmstadt, Germany), whereas acetic acid was from the Aldrich Chemical Company, Inc. (Milwaukee, WI, USA).

### LC-MS/MS analysis

To determine concentrations of 66 oxylipins (Table S1), liquid chromatography (LC) coupled to tandem mass spectrometry (MS/MS) analysis was performed in a randomized order subsequent to extraction. An Agilent UHPLC system Infinity 1290 (Agilent Technologies, CA, USA) was used together with an Agilent 6495 Triple Quadrupole system and Agilent Jet stream electrospray ion source operating in negative ionization mode. The autosampler was kept at 10 °C. Aliquots of 10 μL were injected onto a Waters BEH C18 column (2.1 mm × 150 mm, 1.7-μm particle size) held at 40 °C. The mobile phase consisted of 0.1% acetic acid in MilliQ water (A) and acetonitrile/isopropanol (90/10) (B), and the following gradient was employed: 0.0–3.5 min 10–35% B, 3.5–5.5 min 40% B, 5.5–7.0 min 42% B, 7.0–9.0 min 50% B, 9.0–15.0 min 65% B, 15.0–17.0 min 75% B, 17.0–18.5 min 85% B, 18.5–19.5 min 95% B, 19.5–21 min 10% B, and 21.0–25.0 min 10% B at a constant flow rate of 0.3 mL/min. The dynamic multiple reaction monitoring option was used for all analytes. If applicable, two MS/MS transitions were monitored (Table S2). The most intense transition was used for quantification, whereas the second was used for qualification purposes. Internal standards were assigned to native analytes based on structural similarities and hence retention times (Table S2). Sample concentrations were determined using an 8-point internal standard-based calibration curve for each native analyte (in methanol) and normalized to the wet weight of larvae (presented as ng/g larva).

### Method validation

The method (extraction and analysis) was validated by determining the following parameters: limit of quantification (LOQ), limit of detection (LOD), linearity, matrix effect, precision, accuracy, and bench top stability. A 12-point calibration curve of neat solvent standards (0.41 pg/mL–120 ng/mL) was prepared in methanol and injected in triplicate. Manual integration of the peaks was performed using an in-house script. The analyte methods LOD and LOQ were determined as the lowest amount with peak height above a signal-to-noise (S/N) ratio of 3 and 10, respectively. Linearity was expressed as *R*^2^ for each calibration curve.

To assess the matrix effect (enhancement or suppression) on the instrument response of the analytes, extracted matrix was spiked with analytes at three concentrations (3, 15, and 150 ng/mL) and compared to analytes in methanol. After subtracting the peak areas of an extraction blank from the areas of the analytes in the extracted matrix, the matrix effect for each analyte was calculated by dividing the area of the analyte in the matrix by the area of the neat solvent standard.

Since several of the analytes were endogenous compounds in damselfly larvae, the method precision and accuracy were determined for deuterated internal standards in spiked samples. Damselfly larvae were spiked in triplicate with internal standard mixtures at three concentrations (low, medium or high; see Table S4) on three consecutive days and extracted as described above. Peak areas for each internal standard were normalized against CUDA (recovery standard) and compared to internal standards in methanol. The method precision was calculated as the coefficient of variation (% CV) using the average of the triplicates within 1 day of measurements (intraday precision), and between 3 days of measurements (interday precision). The method accuracy was calculated as the mean value of measured concentrations in extracts compared to methanol within 1 day (intraday accuracy) and between 3 days (interday accuracy).

The bench top stability of each analyte and internal standard was assessed by comparing the peak area of a standard mixture before and after standing at room temperature for 6 h.

### Statistical analysis

Patterns in the data were identified by multivariate analysis performed by principal component analysis (PCA) using SIMCA 14.1 (Umetrics, Umeå, Sweden). Since variables (levels of oxylipins) varied in magnitude, data were scaled to unit variance in the calculated model. PCA extracts the largest sources of variance in the data into principal components. Principal components were plotted against each other to obtain score and loading plots. The position of each sample (larva) in the resulting coordinate system (score plot) was dependent on its oxylipin profile in relation to the other larvae’s oxylipin profiles. Patterns in the data were interpreted using the loading plots. The model was validated from the amount of variance explained (R2X) and its predictive power (Q2X).

Owing to multiple factors that could potentially affect the developmental variability of oxylipins, two sets of general linear models (GLM) were carried out using IBM SPSS Statistics (version 26) to investigate how (i) the larval stage and (ii) the maternal origin affect oxylipin levels. Tukey’s post-hoc test was performed to make multiple pairwise comparisons and determine which specific group means were significantly different (*p* ≤ 0.05). The Bonferroni correction was used to compensate for multiple comparisons. GLM with Tukey’s post-hoc test and the Bonferroni correction were also performed to determine significant differences (*p* ≤ 0.05) in means of the data from the exposure study (unexposed vs. exposed).

A power analysis was performed for one larval stage (L-3) of the developmental and exposure datasets using a web-based program (Power and sample size n.d.). The sample size required to detect existing effects of exposure was calculated for each oxylipin based on the respective standard deviations and differences of means (*p* = 0.05, power 0.8).

## Results and discussion

### Method validation

Usually, extraction protocols for oxylipin analysis involve a solid-phase extraction (SPE) step (Ostermann et al. 2015). We omitted this step for damselfly larvae because it resulted in poorer internal standard recoveries (see Supplementary Information for a comparison of extraction protocols). All analytes displayed good linearity: *R*^2^ values were above 0.99 (0.9923–1) for all calibration curves. LODs ranged from 0.04 to 25 pg on the column and LOQs from 0.14 to 82 pg on the column. The matrix effect was < 100% (3.4–84%) for all analytes, indicating ion-suppressing matrix effects. The benchtop stability over 6 h ranged from 70 to 110% for all analytes and internal standards (Table S3).

The intraday method precision was < 20%, except for 9-HODE-d4 at 230 pg on column (36%), 20-HETE-d6 at 115 pg on column (34%), and 11(12)-EpETrE-d11 at 115 pg on column (27%) (see Table S5). The interday method precision was also < 20%, except for 9-HODE-d4 (51, 49, and 29%, respectively for the three levels tested), PGE2-d4 (26%), TXB2-d4 (27%), PGD2-d4 (25%), 5-HETE-d8 (24%), and 11(12)-EpETrE-d11 (27%) at 230 pg on column, 20-HETE-d6 (35%, 37%, respectively) at 45 and 450 pg on column see Table S5).

The intraday method accuracy was between 39 and 96%, except for 9-HODE-d4 (around 10% at all three tested levels). The interday method accuracy was between 42 and 97%, except for 9-HODE-d4 (around 15% at all three tested levels), leading to limited use of this internal standard (only for 9-HODE and 13-HODE). Both the method precision and accuracy were inferior to previously reported values for the instrument precision and accuracy (Gouveia-Figueira et al. 2015). This was expected since the method included sample extraction, which influences the reported performance parameters. It is likely that omitting SPE had a negative impact on the method performance, but owing to the high number of analytes detected and simpler workflow, this tradeoff was deemed acceptable for practical environmental monitoring programs. However, this emphasizes the need for investigating the analytical variability of the method in relation to the natural variability of the oxylipins under study using the proposed extraction protocol. In order for an oxylipin to function as a useful biomarker for exposure, the analytical variability must be smaller than the natural variability (e.g., developmental variability due to larvae stage and maternal origin, as investigated here). Also, detectable changes in the response to an environmental challenge need to surpass both the analytical and natural variability in a representative and useful sample size.

### Baseline levels of oxylipins detected in damselfly larvae

A total of 38 out of 66 oxylipins probed for (Table S2) were quantified in damselfly larvae at an average concentration between 1.6 (9,10-EpOME) and 2700 (13-HODE) ng per g (wet weight) (Table S6). They derived from four different PUFAs (AA, ALA, EPA, LA), mainly via the CYP and LOX enzymatic pathways. AA metabolites included CYP-derived DiHETrES and EpETrEs and LOX-derived HETEs and oxo-ETEs. ALA derivatives measured were DiHODEs (CYP), EpODEs (CYP) and HOTrEs (LOX). EPA derivatives included DiHETEs (CYP), EpETEs (CYP), and HEPEs (LOX). Oxylipins from LA included CYP-derived DiHOMEs, 9(10)-EpOME, LOX-derived HODEs and 13-oxo-ODE, and the non-enzymatic oxylipin EKODE.

### Variation of oxylipins in damselflies at different developmental stages

**Fig. 2:**
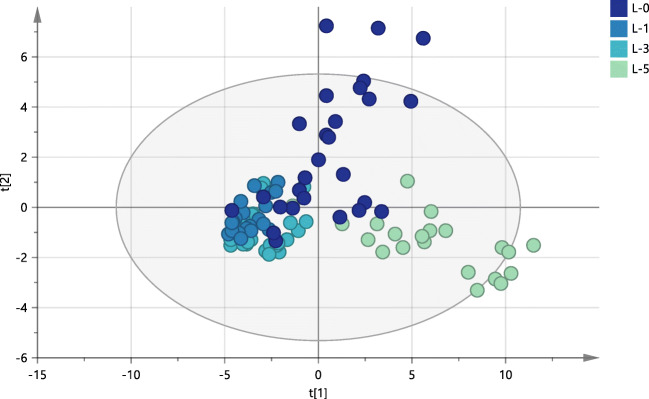
PCA score plot of oxylipins in damselflies showing smaller variation across the larval stage L-3 (turquoise) and L-1 (blue), and larger variation across the larval stage L-5 (green) and adult stage L-0 (dark blue). R2X (cum) = 0.61 and Q2 (cum) = 0.48 for the first two components

Differences and similarities of oxylipin profiles in non-exposed damselflies at different life stages were investigated by PCA. The first two principal components accounted for 49% and 12% of the total variance, respectively. No complete separation was observed between developmental stages (Fig. 2). The two intermediate stages (L-3 and L-1) displayed similar oxylipin profiles and plotted closely together, whereas the earliest stage (L-5) and adult stage (L-0) were more dispersed in the score plot. The loading plot (Figure S1) showed that increased levels of three AA-derived epoxides (8(9)-, 11(12)-, and 14(15)-EpETrE) had the highest influence on the spread of L-0. High levels of another group of compounds (EKODE, 9(10)-EpOME, 9-HOTrE, 5-oxo-ETE, 9,10-DiHOME) were responsible for the spread in L-5. The observed variation was smallest in L-1, followed by L-3.

Variability among the oxylipin levels was in the range of 22–150% (Figure S2a)-d)), as the coefficient of variation (% CV) showed similar ranges across the developmental stages (22–90%, 28–150%, 27–94%, 33–150% for L-5, L-3, L-1, and L-0, respectively) (Figure S2a)-d)). However, fewer oxylipins with lower CVs (< 60%) were observed for the adult stage in comparison to the larval stages (22 for L-0 vs. 32, 29, and 32 for L-5, L-3, and L-1, respectively). To further investigate the ideal stage for sampling with regard to variability, GLM was carried out to assess significant developmental variations in individual oxylipin baseline levels. There were significant developmental variations between all stages. Most oxylipins were significantly different in the stage comparisons (L-5-vs. L-3, L-5 vs. L-1, L-5 vs. L-0, L-3 vs. L-1, L-3 vs. L-0, L-1 vs. L-0) (Table 1). For larval stage comparison, L-3 vs. L-1, only one oxylipin (9,10-DiHOME) was significantly different.

**Table 1:** Significant (marked x) differences in oxylipin baseline levels in damselflies between life stages L-5, L-3, L-2, and L-0 and due to maternal origin (*p* < 0.05).

	Life stage							Maternal origin			
Oxylipin	L5 vs*.* L3	L5 vs*.* L1	L5 vs. L0	L3 vs*.* L1	L3 vs. L0	L1 vs. L0	Number of significant changes	L5	L3	L1	L0
12,13-DiHOME							0				x
12,13-DiHODE							0				x
15,16-DiHODE							0			x	x
14,15-DiHETrE							0				
8,9-DiHETE							0			x	x
14,15-DiHETE							0				x
17,18-DiHETE							0				x
9-HOTrE							0			x	x
8-HETE							0				x
9-HETE							0			x	
9(10)-EpOME							0		x		
14(15)-EpETrE					x	x	2		x		
8(9)-EpETrE			x		x	x	3				
11(12)-EpETrE			x		x	x	3				
15-HEPE	x	x	x			x	4				
15-oxo-ETE	x	x	x			x	4		x	x	x
8,9-DiHETrE	x	x	x				3				x
9-HODE	x	x	x				3				
12-HETE	x	x	x				3				x
5-oxo-ETE	x	x	x				3				
13-oxo-ODE	x	x	x				3		x		x
EKODE	x	x	x				3				
9,10-DiHODE	x	x			x	x	4				x
11,12-DiHETrE	x	x			x	x	4				x
11,12-DiHETE	x	x			x	x	4			x	x
11(12)-EpETE	x	x			x	x	4				
14(15)-EpETE	x	x			x	x	4		x		x
15(16)-EpODE	x	x			x	x	4			x	x
5,6-DiHETrE	x	x	x		x		4				x
9,10-DiHOME	x	x	x	x			4				
13-HODE	x	x	x		x	x	5				
13-HOTrE	x	x	x		x	x	5				x
5-HETE	x	x	x		x	x	5				
11-HETE	x	x	x		x	x	5			x	
15-HETE	x	x	x		x	x	5				x
12-HEPE	x	x	x		x	x	5			x	
9(10)-EpODE	x	x	x		x	x	5				x
12(13)-EpODE	x	x	x		x	x	5			x	

To assess whether the maternal origin of damselflies influenced developmental variations, a second set of GLM was performed. The further along damselflies were in their lifespan, the more oxylipin levels depended on the damselflies’ maternal origin: 0, 5, 10, and 21 oxylipins showed significant differences between larvae of different females in stages L-5, L-3, L-1, and L-0, respectively (Table 1). To avoid large natural variation, knowledge of the larval stage is crucial when sampling for environmental monitoring studies. However, as shown, it is less crucial for larvae at intermediate stages L-3 and L-1, as they display less developmental variation and similar oxylipin baseline levels within the groups.

### Exposure study: oxylipins responsive to wastewater effluent pollutants

**Fig. 3:**
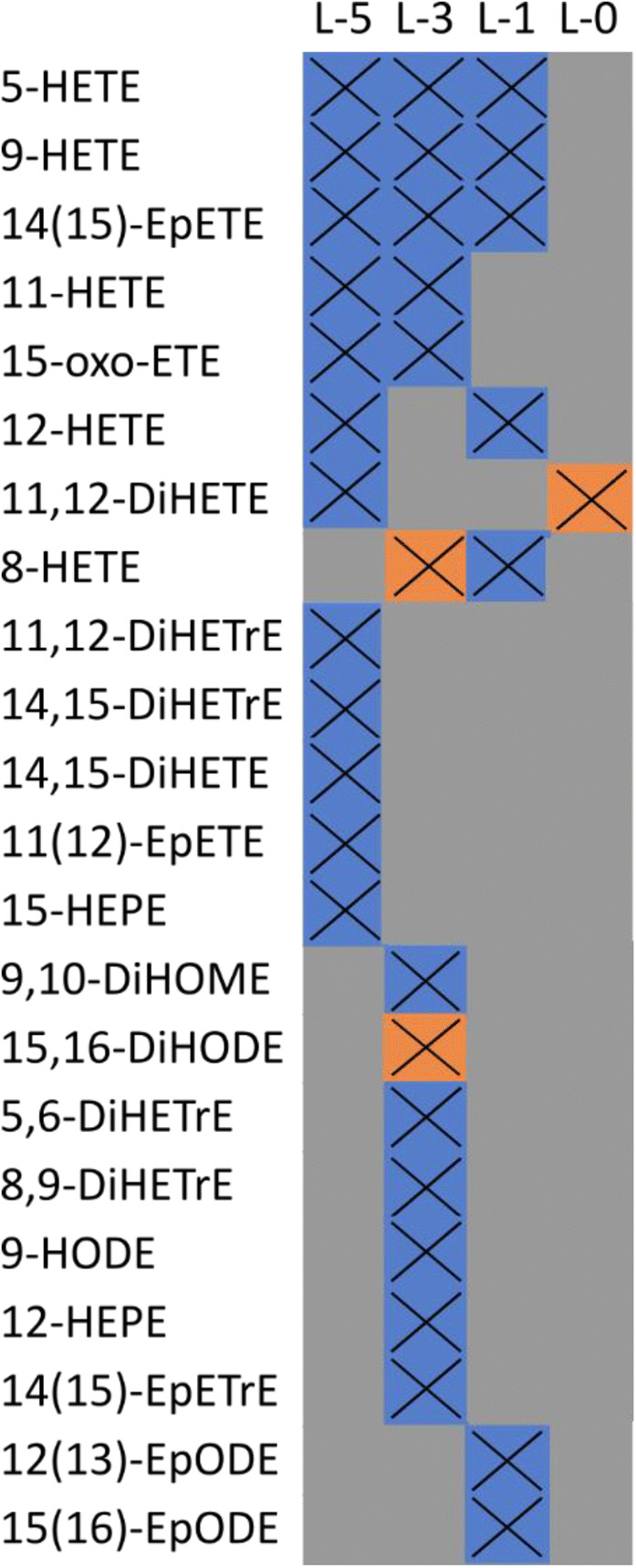
Significant (p < 0.05, marked X) increases (orange) and decreases (blue) of oxylipin levels in damselflies at four developmental stages (L-5, L-3, L-1, and L-0) following wastewater effluent exposure for the duration of one complete larval stage prior to the analyzed one in comparison to non-exposed individuals at the same stage.

GLM was used to investigate significant changes in oxylipin levels (at different stages) of larvae exposed to wastewater effluent for the duration of one larval stage prior to the analyzed one. Out of the 38 detected oxylipins, levels of 22 were responsive to a significant extent to effluent exposure in at least one of the stages; three oxylipins were affected in three stages and five in two stages (*p* < 0.05) (Fig. 3). Most oxylipins were affected in the earliest larval stages L-5 and L-3 (12 and 13, respectively), less in larval stage L-1 (7), and only one oxylipin was affected in the adult stage. Overall, exposure to effluent mainly resulted in decreased oxylipin levels, which is in line with our previous study on oxylipins in damselfly larvae following effluent exposure (Späth et al. 2020). In contrast, 8-HETE and 15,16-DiHODE were increased in larval stage L-3, and 11,12-DiHETE was increased in the adult stage (L-0). It is noteworthy that effluent exposure during the stage preceding, the analyzed stage had no influence on the number of days the larvae spent transitioning from one stage to another (Student’s *t* test, *p* < 0.05, data not shown). Among the three larval stages (L-5, L-3, and L-1), five AA-derived alcohols (5-, 8-, 9-, 11-, and 12-HETE) generated via the LOX pathway played an important role in the exposure response; four of these were affected in each stage. An EPA-derived epoxide 14(15)-EpETE generated via the CYP pathway was suppressed in exposed larvae throughout the three larval stages. Other epoxides deriving from ALA were suppressed in larval stage L-1 (12(13)-, 15(16)-EpODE), and from AA (14(15)-EpETrE) in L-3, respectively. In the two earliest larval stages, several diols generated via the CYP and sEH pathways were also affected (in L-5: 11,12, and 14,15-DiHETE from EPA; 11,12, and 14,15-DiHETrE from AA; in L-3: 9,10-DiHOME from LA; 15,16-DiHODE from ALA; and 5,6 and 8,9-DiHETrE from AA). As mentioned, in the adult stage, only 11,12-DiHETE was responsive to exposure. The interpretation of these findings is challenging since there is limited knowledge of the function of these specific oxylipins in invertebrates. We can only detect a deviation from the normal state, but not interpret the effect of this deviation on the individuals. Therefore at this stage, oxylipins function as class 1 biomarkers, i.e., they show effects of exposure to a pollutant, but no prediction on effect on individuals and the population can be made (Depledge and Fossi 1994).

The extent to which oxylipins were affected by effluent exposure was dependent on the life stage of the individual. In terms of responsiveness of oxylipins, the transition between L-1 to the adult stage appeared to be less affected than earlier stages (e.g., L-5, L-3) by effluent exposure (despite L-1 individuals being exposed for a longer period in comparison to the other stages). Therefore, sampling larvae at early stages is recommended to elucidate effects related to wastewater effluent exposure.

### Recommendations for future use of oxylipins in damselflies as biochemical biomarkers in environmental monitoring programs

Our results from the current study highlight the importance of knowledge and careful selection of developmental stage when sampling an invertebrate species for environmental monitoring studies. To avoid underestimating the effects of pollutant exposure, larvae should be sampled at the same life stage; otherwise, there is a risk of missing effects due to higher developmental variation within different stages in comparison to the variation caused by exposure, as well as due to different exposure responses for different stages. For damselfly *C.H*., larval stage L-3 was found to be the ideal stage for biomarker investigations regarding wastewater effluent pollutants because oxylipin profiles displayed low developmental variance, while a wide range of oxylipins were responsive to exposure. However, in this pilot study of exposure effects, few individuals were included, so other options for larval stage explorations cannot be excluded. The adult stage seemed not suitable for detecting alterations in oxylipin profiles caused by wastewater exposure.

**Fig. 4:**
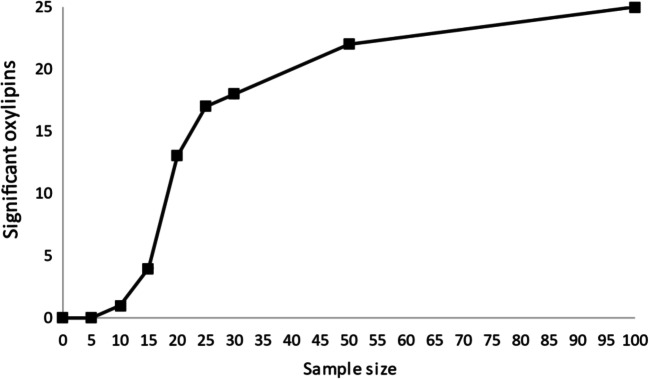
Relationship of group size and number of oxylipins detected as significantly affected by wastewater exposure; calculated by power analysis. Calculations are based on standard deviations of non-exposed damselfly larvae (stage L-3), differences of means (non-exposed vs. exposed), *p* value 0.05, power 0.8, e.g., with a sample size of *n* = 25 (per group), the null hypothesis, that means of exposed and non-exposed groups are equal, can be rejected for 17 oxylipins with a probability of 0.8

The obtained results on the variation of individual oxylipins in damselflies can be used to estimate necessary sample sizes for future experiments. The probability of detecting differences between two populations depends on the variation within populations, the difference in means between populations, and the sample size. The smaller the variation within populations, the larger the difference between them, and the larger the sample size, the more likely existing effects can be detected. As an example, using power analysis, we calculated for all oxylipins at the optimal larval stage (L-3), how many samples were required to likely (power 0.8) detect a significant (*α* = 0.05) effect of effluent exposure. Figure 4 shows how many oxylipins would show a significant effect of exposure depending on the sample size (per group). As there is a steep incline in significant oxylipins up to 25 samples, we recommend a sample size of 25 individuals per group for comparable exposure experiments. Further research is needed to better understand the impact of each altered oxylipin on the organism. When we know the most important oxylipins for this effect, the sample size can be reduced, which will make it more feasible to implement them as biomarkers in environmental monitoring programs.

The strengths of the current study include the well-controlled conditions during the rearing of damselflies in terms of exogenous (e.g., light, temperature, spatial positioning, food availability) and endogenous (e.g., knowledge of species and maternal origin) factors. However, as the requirements for food change over the lifespan (i.e., smaller larvae feed on smaller prey than larger ones), differences in feeding over time could not be avoided. Because the PUFA composition likely differs between pond zooplankton, cultivated zooplankton, and artemia, it is plausible that at least part of the observed variation in oxylipin profiles across different life stages was caused by changes in diet. The laboratory set up has additional limitations with regard to environmental factors, such as region, climate, season, light, and predation pressure, which all contribute to natural variation. Furthermore, larvae were only exposed for the duration of one larval stage, where in real-life exposure through several life stages is expected. Addressing these limitations requires further studies on larvae collected in their natural habitat across different sites.

There is a need to develop new indicators and monitoring schemes for water quality and aquatic ecosystems, especially with regard to the effect from emerging pollutants defined as “chemicals that are not commonly monitored but have the potential to enter the environment and cause adverse ecological and human health effects” (Geissen et al. 2015). Emerging pollutants require new types of methodologies for sampling, detection, and quantification of their effects, with emphasis on monitoring the combined effects of chemicals (i.e., chemical mixtures). Integrative bio-assessment tools and new biomarkers such as oxylipins may offer a suitable approach for addressing these challenges. One concrete example, where this method has the potential to increase both cost-efficiency and generality of the results is the monitoring program for running waters in Sweden—governed by regional county boards. When we better understand the connection between water-quality and oxylipin-response in damselflies (or other aquatic invertebrates), we could, instead of collecting water for chemical analysis and biodiversity-samples for ecosystem health purposes, simply collect a set of aquatic invertebrates and analyze their oxylipin levels. This would save not only time and money, but also several hundred-thousands of aquatic invertebrates yearly. As for the choice of model organism the damselfly *C.H.* is widely distributed across northern and central Europe and Asia, and other similar damselfly species are abundant on every continent except Antarctica, making them good sentinel species for environmental monitoring strategies across the world. Oxylipin analysis is a promising tool that has the potential to be useful in environmental monitoring and assessment, but before oxylipins can be implemented in monitoring programs, increased knowledge regarding their variation across damselfly species, and other aquatic organisms, is needed.

In summary, we investigated oxylipin baseline levels in damselfly *C.H.* and their variations across different life stages in a well-controlled set-up. The results highlight the importance of knowledge about larval stages when sampling for environmental monitoring studies, particularly in this case when determining the suitability of oxylipins in damselfly larvae as biochemical biomarkers of anthropogenic pollution. Future research is needed to pinpoint the most relevant oxylipins to environmental insult and identify their variation in different damselfly species collected in their natural habitat across different sites.

## Supplementary Information

ESM 1 (DOCX 258 kb).

## Data Availability

The datasets used and analyzed during the current study are available from the corresponding author on reasonable request.

## References

[CR1] Boroń M, Mirosławski J (2009). Using insects (Damselflies: Azure Damselfly – Coenagrion Puella) as biomarkers of environmental pollution. Fresenius Environmental Bulletin.

[CR2] Daughton CG, Ternes TA (1999). Pharmaceuticals and personal care products in the environment: agents of subtle change. Environ Health Perspect.

[CR3] David A, Lange A, Abdul-Sada A, Tyler CR, Hill EM (2017). Disruption of the prostaglandin metabolome and characterization of the pharmaceutical exposome in fish exposed to wastewater treatment works effluent as revealed by nanoflow-nanospray mass spectrometry-based metabolomics. Environ Sci Technol.

[CR4] Dennis EA, Norris PC (2015). Eicosanoid storm in infection and inflammation. Nat Rev Immunol.

[CR5] Depledge MH, Fossi MC (1994). The role of biomarkers in environmental assessment (2). Invertebrates. Ecotoxicology.

[CR6] Fleeger JW, Carman KR, Nisbet RM (2003). Indirect effects of contaminants in aquatic ecosystems. Sci Total Environ.

[CR7] Garreta-Lara E, Checa A, Fuchs D, Tauler R, Lacorte S, Wheelock CE, Barata C (2018). Effect of psychiatric drugs on Daphnia magna oxylipin profiles. Sci Total Environ.

[CR8] Geissen V, Mol H, Klumpp E, Umlauf G, Nadal M, van der Ploeg M, van de Zee SEATM, Ritsema CJ (2015). Emerging pollutants in the environment: a challenge for water resource management. Int Soil Water Conserv Res.

[CR9] Gouveia-Figueira S, Späth J, Zivkovic AM, Nording ML (2015). Profiling the oxylipin and endocannabinoid metabolome by UPLC-ESI-MS/MS in human plasma to monitor postprandial inflammation. PLoS ONE.

[CR10] Häder DP, Banaszak AT, Villafañe VE, Narvarte MA, González RA, Helbling EW (2020). Anthropogenic pollution of aquatic ecosystems: emerging problems with global implications. Sci Total Environ.

[CR11] Halling-Sørensen B, Nors Nielsen S, Lanzky PF, Ingerslev F, Holten Lützhøft HC, Jørgensen SE (1998). Occurrence, fate and effects of pharmaceutical substances in the environment- a review. Chemosphere.

[CR12] Heckmann LH, Sibly RM, Timmermans MJTN, Callaghan A (2008). Outlining eicosanoid biosynthesis in the crustacean Daphnia. Front Zool.

[CR13] Janssens L, Stoks R (2013). Fitness effects of chlorpyrifos in the damselfly Enallagma cyathigerum strongly depend upon temperature and food level and can bridge metamorphosis. PLoS ONE.

[CR14] Jonsson M, Fick J, Klaminder J, Brodin T (2014). Antihistamines and aquatic insects: bioconcentration and impacts on behavior in damselfly larvae (Zygoptera). Sci Total Environ.

[CR15] Kidd KA, Blanchfield PJ, Mills KH, Palace VP, Evans RE, Lazorchak JM, Flick RW (2007). Collapse of a fish population after exposure to a synthetic estrogen. Proc Natl Acad Sci.

[CR16] Knight J, Rowley AF, Yamazaki M, Clare AS (1999). Eicosanoids are modulators of larval settlement in the barnacle, Balanus amphitrite. J Mar Biol Assoc U K.

[CR17] Luo Y, Guo W, Ngo HH, Nghiem LD, Hai FI, Zhang J, Liang S, Wang XC (2014). A review on the occurrence of micropollutants in the aquatic environment and their fate and removal during wastewater treatment. Sci Total Environ.

[CR18] Monserrat JM, Martínez PE, Geracitano LA, Amado LL, Martins CMG, Pinho GLL, Chaves IS, Ferreira-Cravo M, Ventura-Lima J, Bianchini A (2007). Pollution biomarkers in estuarine animals: critical review and new perspectives. Comp Biochem Physiol C Toxicol Pharmacol.

[CR19] Norling U (1984). The life cycle and larval photoperiodic responses of Coenagrion hastulatum (Charpentier) in two climatically different areas (Zygoptera: Coenagrionidae). Odonatologica.

[CR20] Ostermann AI, Willenberg I, Schebb NH (2015). Comparison of sample preparation methods for the quantitative analysis of eicosanoids and other oxylipins in plasma by means of LC-MS/MS. Anal Bioanal Chem.

[CR21] Power and sample size (n.d.) https://statcomp2.app.vumc.org/ps/. Accessed April 22, 2020

[CR22] Previšić A, Rožman M, Mor JR, Acuña V, Serra-Compte A, Petrović M, Sabater S (2020). Aquatic macroinvertebrates under stress: bioaccumulation of emerging contaminants and metabolomics implications. Sci Total Environ.

[CR23] Rodrigues C, Guimarães L, Vieira N (2019). Combining biomarker and community approaches using benthic macroinvertebrates can improve the assessment of the ecological status of rivers. Hydrobiologia.

[CR24] Sarkar A, Ray D, Shrivastava AN, Sarker S (2006). Molecular biomarkers: their significance and application in marine pollution monitoring. Ecotoxicology.

[CR25] Scarduelli L, Giacchini R, Parenti P, Migliorati S, Di Brisco AM, Vighi M (2017). Natural variability of biochemical biomarkers in the macro-zoobenthos: dependence on life stage and environmental factors. Environ Toxicol Chem.

[CR26] Späth J, Nording M, Lindberg R, Brodin T, Jansson S, Yang J, Wan D, Hammock B, Fick J (2020). Novel metabolomic method to assess the effect-based removal efficiency of advanced wastewater treatment techniques. Environ Chem.

[CR27] Storhaug E, Nahrgang J, Pedersen KB, Brooks SJ, Petes L, Bakhmet IM, Frantzen M (2019). Seasonal and spatial variations in biomarker baseline levels within Arctic populations of mussels (Mytilus Spp.). Sci Total Environ.

[CR28] van der Oost R, Beyer J, Vermeulen NPE (2003). Fish bioaccumulation and biomarkers in environmental risk assessment: a review. Environ Toxicol Pharmacol.

[CR29] Wigh A, Geffard O, Abbaci K, Francois A, Noury P, Bergé A, Vulliet E (2017). Gammarus fossarum as a sensitive tool to reveal residual toxicity of treated wastewater effluents. Sci Total Environ.

[CR30] Yang J, Schmelzer K, Georgi K, Hammock BD (2009). Quantitative profiling method for oxylipin metabolome by liquid chromatography electrospray ionization tandem mass spectrometry. Anal Chem.

