# Supplementary material for: Oxylipins at intermediate larval stages of damselfly Coenagrion hastulatum as biochemical biomarkers for anthropogenic pollution
Source: Environ Sci Pollut Res Int. 2021 Jan 29;28(22):27629–38. doi: 10.1007/s11356-021-12503-x (PMC8164578; doi:10.1007/s11356-021-12503-x)
Supplement: Supplementary file 1 — (DOCX 258 kb). [file 11356_2021_12503_MOESM1_ESM.docx]

**Supporting Information**

- Tables of analyzed oxylipins (Table S1), MS/MS transitions (Table S2), method validation parameters (Table S3, Table S4, Table S5) and oxylipin levels in samples (Table S6).
- Information on wet weight and exoskeleton length (Table S7).
- Figures of loadings plot of principal component analysis (Figure S1) and coefficients of variance for instars L-5, L-3, L-1, L-0 (Figure S2 a)-d)).
- Description of Umeå wastewater treatment plant: text
- Extraction protocol comparison: text and Figure S3.

**Oxylipins at intermediate larval stages of damselfly *Coenagrion hastulatum* as biochemical biomarkers for anthropogenic pollution**

Jana Späth ^a*^, Tomas Brodin ^b,c^, Daniel Cerveny ^c,d^, Richard Lindberg ^a^, Jerker Fick ^a^, Malin L. Nording ^a^

^a^ Department of Chemistry, Umeå University, SE 90187 Umeå, Sweden

^b^ Department of Ecology and Environmental Science, SE 90187 Umeå University, Sweden

^c^ Department of Wildlife, Fish, and Environmental Studies, Swedish University of Agricultural Sciences, SE 90183 Umea ̊, Sweden

^d^ University of South Bohemia in Ceske Budejovice, Faculty of Fisheries and Protection of Waters, South Bohemian Research Center of Aquaculture and Biodiversity of Hydrocenoses, Zatisi 728/II, Vodnany, Czech Republic

^*^ Corresponding author: jana.spath@umu.se

Number of pages: 24

Number of figures: 6

Number of tables: 6

Table S1. Oxylipin nomenclature, molecular formula, polyunsaturated fatty acid (PUFA) precursor, class, enzymatic pathway, CAS, supplier.

| Oxylipin | Full name | Molecular Formula | PUFA | Class | Enzyme | CAS | Supplier |
| --- | --- | --- | --- | --- | --- | --- | --- |
| 9,10,13-TriHOME | 9,10,13-Trihydroxy-octadecenoic acid | C18H34O5 | LA | Triol | 5-LOX | 29907-57-1 | Larodan, SE, Malmö |
| 9,12,13-TriHOME | 9,12,13-Trihydroxy-octadecenoic acid | C18H34O5 | LA | Triol | 5-LOX | 97134-11-7 | Larodan, SE, Malmö |
| PGF2a | Prostaglandin F2a | C20H34O5 | AA | Triol | COX | 551-11-1 | Cayman Chemical |
| Resolvin D1 | 7,8,17-Trihydroxy-docosahexaenoic acid | C22H32O5 | DHA | Triol | 5,15-LOX | 872993-05-0 | Cayman Chemical |
| Resolvin D2 | 7,16,17-Trihydroxy-docosahexaenoic acid | C22H32O6 | DHA | Triol | 5,15-LOX | 810668-37-2 | Cayman Chemical |
| TXB2 | Thromboxane B2 | C20H34O6 | AA | Triol | COX | 54397-85-2 | Cayman Chemical |
| LTB4 | Leukotriene B4 | C20H32O4 | AA | Diol | 5-LOX | 71160-24-2 | Cayman Chemical |
| PGD2 | Prostaglandin D2 | C20H32O5 | AA | Diol/Ketone | COX | 41598-07-6 | Cayman Chemical |
| PGE2 | Prostaglandin E2 | C20H32O5 | AA | Diol/Ketone | COX | 363-24-6 | Cayman Chemical |
| 9,10-DiHOME | 9,10-Dihydroxy-octadecenoic acid | C18H34O4 | LA | Diol | CYP,sEH | 263399-34-4 | Cayman Chemical |
| 12,13-DiHOME | 12-Hydroxy-eicosapentaenoic acid | C18H34O5 | LA | Diol | CYP,sEH | 263399-35-5 | Cayman Chemical |
| 9,10-DiHODE | 9,10-Dihydroxy-octadecadienoic acid | C18H32O4 | ALA | Diol | CYP,sEH | n/a | In-house synthesis |
| 12,13-DiHODE | 12,13-Dihydroxy-octadecadienoic acid | C18H32O4 | ALA | Diol | CYP,sEH | n/a | In-house synthesis |
| 15,16-DiHODE | 15,16-Dihydroxy-octadecadienoic acid | C18H32O4 | ALA | Diol | CYP,sEH | n/a | In-house synthesis |
| 5,6-DiHETrE | 5,6-Dihydroxy-eicosatrienoic acid | C20H34O4 | AA | Diol | CYP,sEH | 213382-49-1 | Cayman Chemical |
| 8,9-DiHETrE | 8,9-Dihydroxy-eicosatrienoic acid | C20H34O4 | AA | Diol | CYP,sEH | 192461-96-4 | Cayman Chemical |
| 11,12-DiHETrE | 11,12-Dihydroxy-eicosatrienoic acid | C20H34O4 | AA | Diol | CYP,sEH | 192461-95-3 | Cayman Chemical |
| 14,15-DiHETrE | 14,15-Dihydroxy-eicosatrienoic acid | C20H34O4 | AA | Diol | CYP,sEH | n/a | Cayman Chemical |
| 8,9-DiHETE | 8,9-Dihydroxy-eicosatetraenoic acid | C20H32O4 | EPA | Diol | CYP,sEH | 867350-87-6 | Cayman Chemical |
| 11,12-DiHETE | 11,12-Dihydroxy-eicosatetraenoic acid | C20H32O4 | EPA | Diol | CYP,sEH | 867350-92-3 | Cayman Chemical |
| 14,15-DiHETE | 14,15-Dihydroxy-eicosatetraenoic acid | C20H32O4 | EPA | Diol | CYP | n/a | Cayman Chemical |
| 17,18-DiHETE | 17,18-Dihydroxy-eicosatetraenoic acid | C20H32O4 | EPA | Diol | CYP | n/a | Cayman Chemical |
| 7,8-DiHDPE | 7,8-Dihydroxy-docosapentaenoic acid | C22H34O4 | DHA | Diol | CYP | 168111-93-1 | Cayman Chemical |
| 10,11-DiHDPE | 10,11-Dihydroxy-docosapentaenoic acid | C22H34O4 | DHA | Diol | CYP | 1345275-22-0 | Cayman Chemical |
| 13,14-DiHDPE | 13,14-Dihydroxy-docosapentaenoic acid | C22H34O4 | DHA | Diol | CYP | 1345275-24-2 | Cayman Chemical |
| 16,17-DiHDPE | 16,17-Dihydroxy-docosapentaenoic acid | C22H34O4 | DHA | Diol | CYP | 1345275-27-5 | Cayman Chemical |
| 19,20-DiHDPE | 19,20-Dihydroxy-docosapentaenoic acid | C22H34O4 | DHA | Diol | CYP | n/a | Cayman Chemical |
| 9-HODE | 9-Hydroxy-octadecadienoic acid | C18H32O3 | LA | Alcohol | 5-LOX | 73543-67-6 | Cayman Chemical |
| 13-HODE | 13-Hydroxy-octadecadienoic acid | C18H32O3 | LA | Alcohol | 5-LOX | 73804-64-5 | Cayman Chemical |
| 9-HOTrE | 9S-Hydroxy-octadecatrienoic acid | C18H30O3 | ALA | Alcohol | 15-LOX | 89886-42-0 | Cayman Chemical |
| 13-HOTrE | 13-Hydroxy-octadecatrienoic acid | C18H30O3 | ALA | Alcohol | 15-LOX | 87984-82-5 | Cayman Chemical |
| 5-HETE | 5-Hydroxy-eicosatetraenoic acid | C20H32O3 | AA | Alcohol | 5-LOX | 73307-52-5 | Cayman Chemical |
| 8-HETE | 8-Hydroxy-eicosatetraenoic acid | C20H32O3 | AA | Alcohol | LOX | 98462-03-4 | Cayman Chemical |
| 9-HETE | 9-Hydroxy-eicosatetraenoic acid | C20H32O3 | AA | Alcohol | 15-LOX | 79495-85-5 | Cayman Chemical |
| 11-HETE | 11-Hydroxy-eicosatetraenoic acid | C20H32O3 | AA | Alcohol | 15-LOX | 73804-65-6 | Cayman Chemical |
| 12-HETE | 12-Hydroxy-eicosatetraenoic acid | C20H32O3 | AA | Alcohol | 15-LOX | 71030-37-0 | Cayman Chemical |
| 15-HETE | 15-Hydroxy-eicosatetraenoic acid | C20H32O3 | AA | Alcohol | 15-LOX | 73836-87-0 | Cayman Chemical |
| 20-HETE | 20-Hydroxy-eicosatetraenoic acid | C20H32O3 | AA | Alcohol | CYP | 79551-86-3 | Cayman Chemical |
| 5-HEPE | 5-Hydroxy-eicosapentaenoic acid | C20H30O3 | EPA | Alcohol | 15-LOX | 83952-40-3 | Cayman Chemical |
| 12-HEPE | 12,13-Dihydroxy-octadecenoic acid | C20H30O3 | EPA | Alcohol | 15-LOX | 54397-83-0 | Cayman Chemical |
| 15-HEPE | 15-Hydroxy-eicosapentaenoic acid | C20H30O3 | EPA | Alcohol | 15-LOX | 88852-33-9 | Cayman Chemical |
| 17-HDoHE | 17-Hydroxy-docosahexaenoic acid | C22H32O3 | DHA | Alcohol | 15-LOX | 90780-52-2 | Cayman Chemical |
| 15-HETrE | 15-Hydroxy-eicosatrienoic acid | C20H34O3 | DGLA | Alcohol | 15-LOX | 92693-02-2 | Cayman Chemical |
| 5-oxo-ETE | 5-Oxo-eicosatetraenoic acid | C20H30O3 | AA | Ketone | 5-LOX | 106154-18-1 | Cayman Chemical |
| 12-oxo-ETE | 12-Oxo-eicosatetraenoic acid | C20H30O3 | AA | Ketone | 15-LOX | 108437-64-5 | Cayman Chemical |
| 15-oxo-ETE | 15-Oxo-eicosatetraenoic acid | C20H30O3 | AA | Ketone | 15-LOX | 81416-72-0 | Cayman Chemical |
| 9-oxo-ODE | 9-Oxo-10,12-octadecadienoic acid | C18H30O3 | LA | Ketone | 5-LOX | 54232-59-6 | Cayman Chemical |
| 13-oxo-ODE | 13-Oxo-octadecadienoic acid | C18H30O3 | LA | Ketone | 5-LOX | 54739-30-9 | Cayman Chemical |
| 9(10)-EpOME | 9(10)-Epoxy-octadecenoic acid | C18H32O3 | LA | Epoxide | CYP | 61949-82-4 | Cayman Chemical |
| 12(13)-EpOME | 12(13)-Epoxy-octadecenoic acid | C18H32O3 | LA | Epoxide | CYP | n/a | Cayman Chemical |
| EKODE | 12(13)-Epoxy-9-keto-octadecenoic acid | C18H30O4 | LA | Epoxide | Non-enz. | 478931-82-7 | Cayman Chemical |
| 9(10)-EpODE | 9(10)-Epoxy-octadecadienoic acid | C18H30O3 | ALA | Epoxide | CYP | n/a | In-house synthesis |
| 12(13)-EpODE | 12(13)-Epoxy-octadecadienoic acid | C18H30O3 | ALA | Epoxide | CYP | n/a | In-house synthesis |
| 15(16)-EpODE | 15(16)-Epoxy-octadecadienoic acid | C18H30O3 | ALA | Epoxide | CYP | n/a | In-house synthesis |
| 8(9)-EpETrE | 8(9)-Epoxy-eicosatrienoic acid | C20H32O3 | AA | Epoxide | CYP | 81246-85-7 | Cayman Chemical |
| 11(12)-EpETrE | 11(12)-Epoxy-eicosatrienoic acid | C20H32O3 | AA | Epoxide | CYP | 123931-40-8 | Cayman Chemical |
| 14(15)-EpETrE | 14(15)-Epoxy-eicosatrienoic acid | C20H32O3 | AA | Epoxide | CYP | 81276-03-1 | Cayman Chemical |
| 8(9)-EpETE | 8(9)-Epoxy-eicosatetraenoic acid | C20H30O3 | EPA | Epoxide | CYP | 851378-93-3 | Cayman Chemical |
| 11(12)-EpETE | 11(12)-Epoxy-eicosatetraenoic acid | C20H30O3 | EPA | Epoxide | CYP | 504435-15-8 | Cayman Chemical |
| 14(15)-EpETE | 14(15)-Epoxy-eicosatetraenoic acid | C20H30O3 | EPA | Epoxide | CYP | 131339-24-7 | Cayman Chemical |
| 17(18)-EpETE | 17(18)-Epoxy-eicosatetraenoic acid | C20H30O3 | EPA | Epoxide | CYP | n/a | Cayman Chemical |
| 7(8)-EpDPE | 7(8)-Epoxy docosapentaenoic acid | C22H32O3 | DHA | Epoxide | CYP | 895127-66-9 | Cayman Chemical |
| 10(11)-EpDPE | 10(11)-Epoxy docosapentaenoic acid | C22H32O3 | DHA | Epoxide | CYP | 895127-65-8 | Cayman Chemical |
| 13(14)-EpDPE | 13(14)-Epoxy docosapentaenoic acid | C22H32O3 | DHA | Epoxide | CYP | 895127-64-7 | Cayman Chemical |
| 16(17)-EpDPE | 16(17)-Epoxy docosapentaenoic acid | C22H32O3 | DHA | Epoxide | CYP | 155073-46-4 | Cayman Chemical |
| 19(20)-EpDPE | 19(20)-Epoxy docosapentaenoic acid | C22H32O3 | DHA | Epoxide | CYP | n/a | Cayman Chemical |
| 12,13-DiHOME-d4 |  | C18H30D4O4 |  |  |  | n/a | Cayman Chemical |
| 12(13)-EpOME-d4 |  | C18H28D4O3 |  |  |  | n/a | Cayman Chemical |
| 9-HODE-d4 |  | C18H28D4O3 |  |  |  | 890955-25-6 | Cayman Chemical |
| PGE2-d4 |  | C20H28D4O5 |  |  |  | 34210-10-1 | Cayman Chemical |
| TXB2-d4 |  | C20H30D4O6 |  |  |  | 1346112-79-5 | Cayman Chemical |
| PGD2-d4 |  | C20H28D4O5 |  |  |  | 211105-29-2 | Cayman Chemical |
| 5-HETE-d8 |  | C20H24D8O3 |  |  |  | 330796-62-8 | Cayman Chemical |
| 20-HETE-d6 |  | C20H24D8O3 |  |  |  | n/a | Cayman Chemical |
| 11(12)-EpETrE-d11 |  | C20H21D11O3 |  |  |  | n/a | Cayman Chemical |
| CUDA |  | C19H36N2O3 |  |  |  | 479413-68-8 | Cayman Chemical |

Table S2. Mass spectrometry parameters for multiple reaction monitoring transitions. Assigned internal standards (IS), collision energies (CE) and retention times (RT) for all oxylipins (fragmentor voltage: 380 V, cell accelerator voltage: 4 V, for all analytes)

| Oxylipin | IS | Precursor | Product Ion 1 | CE 1  (V) | Product Ion 2 | CE 2  (V) | RT  (min) |
| --- | --- | --- | --- | --- | --- | --- | --- |
| 9,10,13-TriHOME | TXB2-d4 | 329.23 | 171 | 21 | 139.1 | 21 | 8.21 |
| 9,12,13-TriHOME | TXB2-d4 | 329.23 | 211.1 | 21 | 229.1 | 17 | 8.03 |
| PGF2a | PGE2-d4 | 353.23 | 193.3 | 21 | 211 | 21 | 8.23 |
| Resolvin D1 | PGE2-d4 | 375.21 | 215.1 | 17 | 136.3 | 13 | 9.76 |
| Resolvin D2 | PGE2-d4 | 375.21 | 215.1 | 17 | 136.3 | 13 | 8.92 |
| TXB2 | TXB2-d4 | 369.23 | 169.1 | 13 | 195 | 9 | 7.47 |
| LTB4 | PGE2-d4 | 335.22 | 195.1 | 13 | 317.2 | 9 | 12.8 |
| PGD2 | PGD2-d4 | 351.21 | 271.2 | 13 | 315.1 | 5 | 8.96 |
| PGE2 | PGE2-d4 | 351.21 | 271.2 | 13 | 315.1 | 5 | 8.52 |
| 9,10-DiHOME | 12,13-DiHOME-d4 | 313.24 | 201 | 17 | 59.1 | 17 | 13.56 |
| 12,13-DiHOME | 12,13-DiHOME-d4 | 313.24 | 183.2 | 17 | 99 | 25 | 13.09 |
| 9,10-DiHODE | 12,13-DiHOME-d4 | 311.22 | 201 | 18 | 275.2 | 14 | 11.85 |
| 12,13-DiHODE | 12,13-DiHOME-d4 | 311.22 | 183.2 | 22 | 293.3 | 18 | 11.82 |
| 15,16-DiHODE | 12,13-DiHOME-d4 | 311.22 | 223.2 | 18 |  |  | 11.77 |
| 5,6-DiHETrE | 20-HETE-d6 | 337.24 | 71 | 25 | 59.1 | 29 | 15.94 |
| 8,9-DiHETrE | 20-HETE-d6 | 337.24 | 127.2 | 21 |  |  | 15.12 |
| 11,12-DiHETrE | 20-HETE-d6 | 337.24 | 167.1 | 17 |  |  | 14.56 |
| 14,15-DiHETrE | 20-HETE-d6 | 337.24 | 207 | 13 | 129.2 | 17 | 13.91 |
| 8,9-DiHETE | 12,13-DiHOME-d4 | 335.22 | 185 | 14 | 127.1 | 26 | 13.32 |
| 11,12-DiHETE | 12,13-DiHOME-d4 | 335.22 | 167.1 | 18 | 149 | 26 | 12.93 |
| 14,15-DiHETE | 12,13-DiHOME-d4 | 335.22 | 207.1 | 18 | 317.1 | 14 | 12.7 |
| 17,18-DiHETE | 12,13-DiHOME-d4 | 335.22 | 247.2 | 14 | 87 | 18 | 12.27 |
| 7,8-DiHDPE | 20-HETE-d6 | 361.24 | 113.1 | 18 | 127 | 14 | 15.57 |
| 10,11-DiHDPE | 20-HETE-d6 | 361.24 | 153.2 | 18 | 149.1 | 14 | 14.86 |
| 13,14-DiHDPE | 20-HETE-d6 | 361.24 | 193.1 | 14 | 343.4 | 14 | 14.53 |
| 16,17-DiHDPE | 20-HETE-d6 | 361.24 | 233.1 | 14 | 343.4 | 14 | 14.3 |
| 19,20-DiHDPE | 20-HETE-d6 | 361.24 | 273.1 | 14 | 229.2 | 18 | 13.87 |
| 9-HODE | 9-HODE-d4 | 295.23 | 171.2 | 13 | 277.2 | 13 | 16.69 |
| 13-HODE | 9-HODE-d4 | 295.23 | 195.1 | 17 | 277.1 | 17 | 16.49 |
| 9-HOTrE | 20-HETE-d6 | 293.21 | 171 | 14 | 275.1 | 14 | 14.89 |
| 13-HOTrE | 20-HETE-d6 | 293.21 | 195.1 | 14 | 275.1 | 14 | 15.07 |
| 5-HETE | 5-HETE-d8 | 319.23 | 257.2 | 9 | 115.1 | 13 | 18.16 |
| 8-HETE | 5-HETE-d8 | 319.23 | 155 | 9 | 301.2 | 9 | 17.67 |
| 9-HETE | 5-HETE-d8 | 319.23 | 167.2 | 9 | 123.1 | 17 | 17.86 |
| 11-HETE | 20-HETE-d6 | 319.23 | 167.2 | 9 |  |  | 17.33 |
| 12-HETE | 5-HETE-d8 | 319.23 | 179.1 | 9 | 301.2 | 9 | 17.58 |
| 15-HETE | 20-HETE-d6 | 319.23 | 301.2 | 9 | 219 | 9 | 16.89 |
| 20-HETE | 20-HETE-d6 | 319.23 | 289.2 | 13 | 180.1 | 13 | 15.55 |
| 5-HEPE | 20-HETE-d6 | 317.21 | 299 | 6 | 255.2 | 10 | 16.51 |
| 12-HEPE | 20-HETE-d6 | 317.21 | 299 | 6 | 179.1 | 9 | 15.96 |
| 15-HEPE | 20-HETE-d6 | 317.21 | 299.2 | 10 | 255.2 | 10 | 15.52 |
| 17-HDoHE | 5-HETE-d8 | 343.23 | 281.2 | 9 | 201.3 | 9 | 16.96 |
| 15-HETrE | 5-HETE-d8 | 321.24 | 303.3 | 9 | 221.1 | 13 | 17.76 |
| 5-oxo-ETE | 5-HETE-d8 | 317.21 | 203.2 | 13 | 59.1 | 21 | 18.81 |
| 12-oxo-ETE | 5-HETE-d8 | 317.21 | 153.2 | 13 | 273.1 | 9 | 17.81 |
| 15-oxo-ETE | 5-HETE-d8 | 317.21 | 113.2 | 13 | 273.1 | 9 | 17.24 |
| 9-oxo-ODE | 20-HETE-d6 | 293.21 | 181.1 | 9 | 97.1 | 25 | 15.06 |
| 13-oxo-ODE | 20-HETE-d6 | 293.21 | 113.1 | 5 | 182.3 | 5 | 16.97 |
| 9(10)-EpOME | 12(13)-EpOME-d4 | 295.23 | 171.2 | 13 |  |  | 18.56 |
| 12(13)-EpOME | 12(13)-EpOME-d4 | 295.23 | 195.1 | 13 | 277.2 | 13 | 18.36 |
| EKODE | 20-HETE-d6 | 309.2 | 291 | 10 | 209.1 | 10 | 15 |
| 9(10)-EpODE | 5-HETE-d8 | 293.21 | 275.2 | 14 | 171.2 | 10 | 16.97 |
| 12(13)-EpODE | 5-HETE-d8 | 293.21 | 183 | 18 | 275.2 | 14 | 17.2 |
| 15(16)-EpODE | 5-HETE-d8 | 293.21 | 235.2 | 10 | 275.2 | 14 | 16.79 |
| 8(9)-EpETrE | 11(12)-EpETrE-d11 | 319.23 | 123 | 5 | 69.2 | 13 | 19.02 |
| 11(12)-EpETrE | 11(12)-EpETrE-d11 | 319.23 | 301.1 | 5 | 167.1 | 9 | 18.86 |
| 14(15)-EpETrE | 11(12)-EpETrE-d11 | 319.23 | 301 | 5 | 219 | 5 | 18.41 |
| 8(9)-EpETE | 5-HETE-d8 | 317.21 | 299.3 | 6 | 255.2 | 10 | 17.64 |
| 11(12)-EpETE | 5-HETE-d8 | 317.21 | 167.1 | 10 | 299.3 | 6 | 17.47 |
| 14(15)-EpETE | 5-HETE-d8 | 317.21 | 207.1 | 10 | 299.3 | 6 | 17.34 |
| 17(18)-EpETE | 5-HETE-d8 | 317.21 | 299.1 | 6 | 259.2 | 10 | 16.87 |
| 7(8)-EpDPE | 12(13)-EpOME-d4 | 343.23 | 281.1 | 6 | 189.1 | 10 | 18.88 |
| 10(11)-EpDPE | 12(13)-EpOME-d4 | 343.23 | 153.1 | 14 | 281.2 | 6 | 18.69 |
| 13(14)-EpDPE | 12(13)-EpOME-d4 | 343.23 | 193.1 | 10 | 281.2 | 6 | 18.59 |
| 16(17)-EpDPE | 12(13)-EpOME-d4 | 343.23 | 281.2 | 6 | 325.3 | 6 | 18.5 |
| 19(20)-EpDPE | 12(13)-EpOME-d4 | 343.23 | 281.2 | 6 | 299.2 | 6 | 18.15 |
| 12,13-DiHOME-d4 |  | 317.26 | 185.1 | 21 |  |  | 13.02 |
| 12(13)-EpOME-d4 |  | 299.25 | 281 | 4 |  |  | 18.29 |
| 9-HODE-d4 |  | 299.25 | 172 | 29 |  |  | 16.61 |
| PGE2-d4 |  | 355.24 | 319.2 | 5 | 275.2 | 13 | 8.49 |
| TXB2-d4 |  | 373.25 | 173 | 9 | 199 | 9 | 7.45 |
| PGD2-d4 |  | 355.24 | 319.2 | 10 | 275.1 | 18 | 8.92 |
| 5-HETE-d8 |  | 327.2 | 116 | 20 |  |  | 18.06 |
| 20-HETE-d6 |  | 325 | 295 | 20 | 279 | 20 | 15.5 |
| 11(12)-EpETrE-d11 |  | 330.29 | 312.2 | 10 | 179.1 | 10 | 18.77 |
| CUDA |  | 339.26 | 214.1 | 17 | 240.2 | 13 | 12.8 |

Table S3. Oxylipin method validation parameters: linearity (R^2^, slope), limit of quantification (LOQ), matrix effect and bench top stability.

| Oxylipin | *R*^2^ | Slope | LOD  (pg on column) | LOQ  (pg on column) | Matrix effect (%)  low | medium | high | Bench top stability (%) |
| --- | --- | --- | --- | --- | --- | --- | --- | --- |
| 9,10,13-TriHOME | 0.9998 | 0.00136 | 0.61 | 2.0 | 52 | 46 | 47 | 96 |
| 9,12,13-TriHOME | 0.9998 | 0.00137 | 0.21 | 0.68 | 55 | 49 | 44 | 100 |
| PGF2a | 0.9999 | 3.3E-05 | 0.61 | 2.04 | 44 | 44 | 46 | 100 |
| Resolvin D1 | 0.9989 | 6.8E-05 | 0.21 | 0.68 | 48 | 43 | 47 | 70 |
| Resolvin D2 | 0.9993 | 1.8E-05 | 12.3 | 40.9 | 23 | 39 | 51 | 70 |
| TXB2 | 0.9995 | 0.00125 | 0.21 | 0.68 | 42 | 44 | 44 | 100 |
| LTB4 | 0.9992 | 0.00013 | 0.21 | 0.68 | 44 | 42 | 40 | 100 |
| PGD2 | 0.9986 | 0.00029 | 0.61 | 2.0 | 38 | 41 | 37 | 94 |
| PGE2 | 0.9995 | 0.00019 | 0.61 | 2.0 | 40 | 44 | 43 | 97 |
| 9,10-DiHOME | 0.9995 | 0.00087 | 0.21 | 0.68 | 45 | 43 | 36 | 98 |
| 12,13-DiHOME | 0.9992 | 0.00049 | 0.21 | 0.68 | 46 | 43 | 47 | 100 |
| 9,10-DiHODE | 0.9999 | 0.00082 | 0.04 | 0.14 | 41 | 48 | 44 | 99 |
| 12,13-DiHODE | 0.9991 | 0.00035 | 0.21 | 0.68 | 45 | 50 | 47 | 98 |
| 15,16-DiHODE | 0.9992 | 0.00017 | 0.61 | 2.0 | 60 | 52 | 46 | 98 |
| 5,6-DiHETrE | 0.9991 | 0.00051 | 0.61 | 2.0 | 37 | 24 | 23 | 99 |
| 8,9-DiHETrE | 0.9991 | 0.00146 | 0.21 | 0.68 | 11 | 36 | 31 | 100 |
| 11,12-DiHETrE | 0.9983 | 0.00376 | 0.21 | 0.68 | 41 | 48 | 28 | 100 |
| 14,15-DiHETrE | 0.998 | 0.0048 | 0.21 | 0.68 | 54 | 48 | 19 | 100 |
| 8,9-DiHETE | 0.9989 | 0.00022 | 0.21 | 0.68 | 48 | 49 | 45 | 99 |
| 11,12-DiHETE | 0.9984 | 0.0008 | 0.04 | 0.14 | 59 | 51 | 47 | 99 |
| 14,15-DiHETE | 0.9995 | 0.00094 | 0.04 | 0.14 | 84 | 71 | 58 | 100 |
| 17,18-DiHETE | 0.9995 | 0.00075 | 0.21 | 0.68 | 45 | 48 | 48 | 99 |
| 7,8-DiHDPE | 1 | 0.00057 | 0.61 | 2.0 | 41 | 36 | 38 | 98 |
| 10,11-DiHDPE | 0.9961 | 0.00374 | 0.21 | 0.68 | 20 | 15 | 18 | 100 |
| 13,14-DiHDPE | 0.9981 | 0.00315 | 0.21 | 0.68 | 29 | 50 | 33 | 100 |
| 16,17-DiHDPE | 0.9979 | 0.00365 | 0.21 | 0.68 | 18 | 32 | 46 | 100 |
| 19,20-DiHDPE | 0.9976 | 0.00268 | 0.21 | 0.68 | 51 | 53 | 27 | 99 |
| 9-HODE | 0.9996 | 0.00174 | 0.04 | 0.14 | n/a | 29 | 21 | 100 |
| 13-HODE | 0.999 | 0.00215 | 0.04 | 0.14 | n/a | 32 | 19 | 100 |
| 9-HOTrE | 0.9996 | 0.00246 | 0.04 | 0.14 | n/a | 6.9 | 15 | 99 |
| 13-HOTrE | 0.9989 | 0.00108 | 0.21 | 0.68 | n/a | 37 | 29 | 96 |
| 5-HETE | 0.9983 | 0.00278 | 0.21 | 0.68 | n/a | 4.1 | 16 | 100 |
| 8-HETE | 0.9997 | 0.00162 | 0.21 | 0.68 | n/a | 71 | 15 | 99 |
| 9-HETE | 0.9999 | 0.00049 | 0.21 | 0.68 | n/a | 28 | 24 | 100 |
| 11-HETE | 0.9992 | 0.00405 | 0.21 | 0.68 | 31 | 36 | 31 | 110 |
| 12-HETE | 0.9988 | 0.00168 | 0.21 | 0.68 | n/a | 37 | 30 | 100 |
| 15-HETE | 0.9994 | 0.00194 | 0.21 | 0.68 | n/a | 45 | 39 | 100 |
| 20-HETE | 0.9966 | 0.00196 | 0.21 | 0.68 | 34 | 36 | 32 | 100 |
| 5-HEPE | 0.9986 | 0.0102 | 0.61 | 2.0 | n/a | 15 | 8.6 | 99 |
| 12-HEPE | 0.9995 | 0.00171 | 0.21 | 0.68 | n/a | n/a | 11 | 96 |
| 15-HEPE | 0.9984 | 0.00361 | 0.61 | 2.04 | n/a | n/a | 31 | 100 |
| 17-HDoHE | 1 | 0.00091 | 0.61 | 2.04 | 47 | 47 | 34 | 100 |
| 15-HETrE | 0.999 | 0.00923 | 0.21 | 0.68 | 27 | 39 | 23 | 100 |
| 5-oxo-ETE | 0.9998 | 0.00046 | 0.61 | 2.04 | 6.8 | 26 | 27 | 85 |
| 12-oxo-ETE | 0.9999 | 0.00044 | 1.2 | 4.1 | 11 | 39 | 25 | 78 |
| 15-oxo-ETE | 0.9995 | 0.00156 | 0.21 | 0.68 | 32 | 45 | 35 | 90 |
| 9-oxo-ODE | 0.9973 | 2.4E-06 | 25 | 82 | n/a | n/a | n/a | 97 |
| 13-oxo-ODE | 0.9997 | 4.1E-05 | 1.23 | 4.10 | 70 | 36 | 30 | 96 |
| 9(10)-EpOME | 0.9997 | 0.00403 | 0.04 | 0.14 | 15 | 16 | 13 | 100 |
| 12(13)-EpOME | 0.9999 | 0.00196 | 0.04 | 0.14 | 15 | 17 | 15 | 96 |
| EKODE | 0.9988 | 0.00721 | 0.04 | 0.14 | 22 | 29 | 30 | 97 |
| 9(10)-EpODE | 0.9963 | 0.00348 | 0.04 | 0.14 | 35 | 33 | 27 | 100 |
| 12(13)-EpODE | 0.9997 | 0.00121 | 0.21 | 0.68 | 26 | 39 | 23 | 99 |
| 15(16)-EpODE | 0.9991 | 0.00237 | 0.21 | 0.68 | 39 | 28 | 24 | 100 |
| 8(9)-EpETrE | 0.9999 | 0.00014 | 0.61 | 2.04 | 6.3 | 6.8 | 7.9 | 98 |
| 11(12)-EpETrE | 0.9923 | 0.00138 | 0.21 | 0.68 | 8 | 11 | 12 | 110 |
| 14(15)-EpETrE | 1 | 0.00062 | 0.61 | 2.0 | 11 | 14 | 13 | 98 |
| 8(9)-EpETE | 0.9999 | 0.00138 | 1.2 | 4.09 | 71 | 38 | 23 | 98 |
| 11(12)-EpETE | 0.9999 | 0.00182 | 0.21 | 0.68 | 39 | 29 | 28 | 100 |
| 14(15)-EpETE | 0.9995 | 0.00188 | 0.21 | 0.68 | 40 | 37 | 29 | 100 |
| 17(18)-EpETE | 0.996 | 0.06961 | 0.04 | 0.14 | 38 | 30 | 31 | 99 |
| 7(8)-EpDPE | 0.9996 | 0.00357 | 0.21 | 0.68 | 3.4 | 8.1 | 10 | 100 |
| 10(11)-EpDPE | 0.9996 | 0.00333 | 0.21 | 0.68 | 26 | 32 | 31 | 100 |
| 13(14)-EpDPE | 0.9998 | 0.00167 | 0.21 | 0.68 | 31 | 31 | 23 | 96 |
| 16(17)-EpDPE | 0.9997 | 0.00119 | 1.23 | 4.1 | 14 | 13 | 15 | 100 |
| 19(20)-EpDPE | 0.9995 | 0.00321 | 0.21 | 0.68 | 39 | 33 | 29 | 95 |

Table S4. Method precision and accuracy: levels of internal standards spiked (ng) and resulting pg on column.

|  | low |  | medium |  | high |  |
| --- | --- | --- | --- | --- | --- | --- |
| Internal standard | ng spiked | pg on column | ng spiked | pg on column | ng spiked | pg on column |
| 12,13-DiHOME-d4 | 1 | 45 | 5 | 225 | 10 | 450 |
| 12(13)-EpOME-d4 | 2 | 91 | 10 | 455 | 20 | 910 |
| 9-HODE-d4 | 0.5 | 23 | 2.5 | 115 | 5 | 230 |
| PGE2-d4 | 0.5 | 23 | 2.5 | 115 | 5 | 230 |
| TXB2-d4 | 0.5 | 23 | 2.5 | 115 | 5 | 230 |
| PGD2-d4 | 0.5 | 23 | 2.5 | 115 | 5 | 230 |
| 5-HETE-d8 | 0.5 | 23 | 2.5 | 115 | 5 | 230 |
| 20-HETE-d6 | 1 | 45 | 5 | 225 | 10 | 450 |
| 11(12)-EpETrE-d11 | 0.5 | 23 | 2.5 | 115 | 5 | 230 |

Table S5. Interday and intraday method precision (coefficient of variation) and accuracy at three concentration levels.

|  | Precision [%] | | | | | | Accuracy [%] | | | | | |
| --- | --- | --- | --- | --- | --- | --- | --- | --- | --- | --- | --- | --- |
|  | intraday | | | interday | | | Intraday | | | Interday | | |
| Internal standard | low | medium | high | low | medium | high | low | medium | high | low | medium | high |
| 12,13-DiHOME-d4 | 8.2 | 5.5 | 5.4 | 14 | 6.6 | 9.9 | 96 | 82 | 91 | 86 | 97 | 71 |
| 12(13)-EpOME-d4 | 14 | 18 | 6 | 10 | 5.3 | 9.2 | 53 | 44 | 50 | 59 | 52 | 43 |
| 9-HODE-d4 | 7.2 | 14 | 36 | 51 | 49 | 29 | 10 | 13 | 11 | 17 | 14 | 14 |
| PGE2-d4 | 6.6 | 0.51 | 4.4 | 17 | 13 | 26 | 79 | 74 | 77 | 69 | 83 | 75 |
| TXB2-d4 | 5.4 | 4 | 3 | 7.7 | 12 | 27 | 81 | 75 | 77 | 77 | 87 | 75 |
| PGD2-d4 | 10 | 4.3 | 3.7 | 15 | 19 | 25 | 55 | 54 | 54 | 48 | 61 | 54 |
| 5-HETE-d8 | 16 | 14 | 3.7 | 15 | 11 | 24 | 61 | 56 | 56 | 77 | 64 | 49 |
| 20-HETE-d6 | 9.1 | 34 | 9.1 | 35 | 17 | 37 | 53 | 39 | 46 | 49 | 51 | 42 |
| 11(12)-EpETrE-d11 | 9.3 | 27 | 1.6 | 13 | 14 | 27 | 62 | 53 | 53 | 84 | 65 | 48 |

Table S6. Average concentrations (ng/g) of oxylipins in damselfly larvae at four instars (L-5, L-3, L-1, L-0).

| PUFA | Enzyme | Oxylipin | L-5 ± 1 | L-3 ± 1 | L-1 | L-0 |
| --- | --- | --- | --- | --- | --- | --- |
| AA | CYP | 5,6-DiHETrE | 110 ± 53 | 35 ± 19 | 19 ± 8 | 73 ± 36 |
|  |  | 8,9-DiHETrE | 47 ± 23 | 14 ± 5.7 | 13 ± 6.6 | 16 ± 12 |
|  |  | 11,12-DiHETrE | 60 ± 30 | 21 ± 10 | 27 ± 16 | 46 ± 32 |
|  |  | 14,15-DiHETrE | 49 ± 18 | 25 ± 13 | 22 ± 14 | 43 ± 68 |
|  |  | 8(9)-EpETrE | 31 ± 15 | 14 ± 7.7 | 9.4 ± 6.3 | 65 ± 72 |
|  |  | 11(12)-EpETrE | 18 ± 7.4 | 6.8 ± 2.3 | 4.1 ± 1.8 | 38 ± 41 |
|  |  | 14(15)-EpETrE | 21 ± 11 | 6.5 ± 3.5 | 3.8 ± 2.2 | 37 ± 54 |
|  | LOX | 5-HETE | 650 ± 290 | 210 ± 98 | 130 ± 40 | 350 ± 130 |
|  |  | 8-HETE | 1400 ± 670 | 1100 ± 380 | 1600 ± 490 | 1300 ± 560 |
|  |  | 9-HETE | 860 ± 450 | 230 ± 110 | 140 ± 66 | 310 ± 170 |
|  |  | 11-HETE | 590 ± 300 | 190 ± 79 | 140 ± 57 | 390 ± 210 |
|  |  | 12-HETE | 1100 ± 510 | 360 ± 130 | 390 ± 120 | 540 ± 250 |
|  |  | 15-HETE | 990 ± 420 | 320 ± 120 | 260 ± 76 | 630 ± 320 |
|  |  | 5-oxo-ETE | 73 ± 42 | 22 ± 14 | 10 ± 3.8 | 16 ± 10 |
|  |  | 15-oxo-ETE | 340 ± 180 | 120 ± 74 | 73 ± 35 | 190 ± 110 |
| ALA | CYP | 9,10-DiHODE | 160 ± 71 | 55 ± 30 | 54 ± 14 | 130 ± 51 |
|  |  | 12,13-DiHODE | 69 ± 31 | 29 ± 21 | 42 ± 30 | 61 ± 43 |
|  |  | 15,16-DiHODE | 480 ± 190 | 210 ± 120 | 300 ± 100 | 480 ± 330 |
|  |  | 9(10)-EpODE | 180 ± 130 | 41 ± 44 | 27 ± 9.6 | 100 ± 81 |
|  |  | 12(13)-EpODE | 68 ± 45 | 18 ± 20 | 11 ± 3.6 | 42 ± 27 |
|  |  | 15(16)-EpODE | 120 ± 85 | 37 ± 42 | 26 ± 9.5 | 83 ± 66 |
|  | LOX | 9-HOTrE | 1100 ± 640 | 170 ± 77 | 110 ± 45 | 190 ± 90 |
|  |  | 13-HOTrE | 2200 ± 1100 | 360 ± 180 | 340 ± 110 | 790 ± 420 |
| EPA | CYP | 8,9-DiHETE | 120 ± 51 | 42 ± 16 | 32 ± 12 | 45 ± 16 |
|  |  | 11,12-DiHETE | 82 ± 28 | 41 ± 18 | 45 ± 16 | 67 ± 24 |
|  |  | 14,15-DiHETE | 67 ± 22 | 39 ± 21 | 43 ± 18 | 57 ± 31 |
|  |  | 17,18-DiHETE | 81 ± 21 | 56 ± 24 | 69 ± 21 | 100 ± 65 |
|  |  | 11(12)-EpETE | 31 ± 12 | 12 ± 5.9 | 6.6 ± 3.1 | 38 ± 22 |
|  |  | 14(15)-EpETE | 38 ± 17 | 16 ± 7.4 | 9.3 ± 2.8 | 37 ± 16 |
|  | LOX | 12-HEPE | 2400 ± 1000 | 940 ± 380 | 700 ± 190 | 1400 ± 540 |
|  |  | 15-HEPE | 820 ± 690 | 150 ± 230 | 75 ± 67 | 410 ± 440 |
| LA | CYP | 9,10-DiHOME | 81 ± 31 | 28 ± 8.1 | 14 ± 5.9 | 19 ± 13 |
|  |  | 12,13-DiHOME | 54 ± 18 | 23 ± 10 | 19 ± 7 | 28 ± 16 |
|  |  | 9(10)-EpOME | 21 ± 17 | 5.8 ± 5.6 | 2 ± 1.6 | 8.7 ± 5.4 |
|  | LOX | 9-HODE | 3600 ± 1500 | 1000 ± 290 | 600 ± 290 | 1100 ± 610 |
|  |  | 13-HODE | 6500 ± 2700 | 2000 ± 720 | 1600 ± 530 | 4100 ± 2300 |
|  |  | 13-oxo-ODE | 880 ± 380 | 260 ± 110 | 160 ± 77 | 270 ± 140 |
|  | Non-enz. | EKODE | 110 ± 77 | 21 ± 22 | 7.9 ± 7.8 | 5.5 ± 3.4 |

Table S7. Wet weight and exoskeleton length of individual damselflies.

| Individual  # | Mother | Larval stage | Exposure | Wet weight (g) | Exoskeleton length  (cm) |
| --- | --- | --- | --- | --- | --- |
| 1 | M1 | L-5 | non-exp | 0.0035 | n/a |
| 2 | M1 | L-5 | non-exp | 0.0047 | n/a |
| 3 | M1 | L-5 | non-exp | 0.0055 | n/a |
| *4* | M1 | L-5 | non-exp | 0.0029 | n/a |
| *5* | M1 | L-5 | non-exp | 0.0043 | n/a |
| *6* | M1 | L-5 | non-exp | 0.0046 | n/a |
| *7* | M1 | L-3 | non-exp | 0.011 | 1.189367 |
| *8* | M1 | L-3 | non-exp | 0.008 | 1.003198 |
| *9* | M1 | L-3 | non-exp | 0.024 | 1.306388 |
| *10* | M1 | L-3 | non-exp | 0.0121 | 1.187425 |
| *11* | M1 | L-3 | non-exp | 0.0101 | 1.032115 |
| *12* | M1 | L-3 | non-exp | 0.0116 | 1.204809 |
| *13* | M1 | L-1 | non-exp | 0.0372 | 1.616457 |
| *14* | M1 | L-1 | non-exp | 0.0335 | 1.530723 |
| *15* | M1 | L-1 | non-exp | 0.0483 | 1.841312 |
| *16* | M1 | L-1 | non-exp | 0.0318 | 1.556327 |
| *17* | M1 | L-1 | non-exp | 0.0355 | 1.783487 |
| *18* | M1 | L-1 | non-exp | 0.0336 | 1.588615 |
| *19* | M1 | L-0 | non-exp | 0.0411 | 3.218021 |
| *20* | M1 | L-0 | non-exp | 0.0293 | 3.284774 |
| *21* | M1 | L-0 | non-exp | 0.0357 | 3.059366 |
| *22* | M1 | L-0 | non-exp | 0.0373 | 3.285787 |
| *23* | M1 | L-0 | non-exp | 0.0408 | 3.266417 |
| *24* | M1 | L-0 | non-exp | 0.053 | 3.32466 |
| *25* | M2 | L-5 | non-exp | 0.004 | n/a |
| *26* | M2 | L-5 | non-exp | 0.0059 | n/a |
| *27* | M2 | L-5 | non-exp | 0.003 | n/a |
| *28* | M2 | L-5 | non-exp | 0.005 | n/a |
| *29* | M2 | L-5 | non-exp | 0.0046 | n/a |
| *30* | M2 | L-5 | non-exp | 0.0068 | n/a |
| *31* | M2 | L-3 | non-exp | 0.0078 | 1.073327 |
| *32* | M2 | L-3 | non-exp | 0.0081 | 1.383042 |
| *33* | M2 | L-3 | non-exp | 0.0096 | 1.162935 |
| *34* | M2 | L-3 | non-exp | 0.0075 | 1.040108 |
| *35* | M2 | L-3 | non-exp | 0.0084 | 0.989952 |
| *36* | M2 | L-3 | non-exp | 0.0099 | 0.749998 |
| *37* | M2 | L-1 | non-exp | 0.0378 | 1.701608 |
| *38* | M2 | L-1 | non-exp | 0.0351 | 1.602132 |
| *39* | M2 | L-1 | non-exp | 0.0308 | 1.605898 |
| *40* | M2 | L-1 | non-exp | 0.0391 | 1.618269 |
| *41* | M2 | L-1 | non-exp | 0.0233 | 1.395447 |
| *42* | M2 | L-1 | non-exp | 0.0275 | 1.421953 |
| *43* | M2 | L-0 | non-exp | 0.0435 | 3.698244 |
| *44* | M2 | L-0 | non-exp | 0.035 | 3.110095 |
| *45* | M2 | L-0 | non-exp | 0.0463 | 3.4523 |
| *46* | M2 | L-0 | non-exp | 0.035 | 3.312946 |
| *47* | M2 | L-0 | non-exp | 0.0326 | 3.366208 |
| *48* | M2 | L-0 | non-exp | 0.0388 | 3.293182 |
| *49* | M3 | L-5 | non-exp | 0.0054 | n/a |
| *50* | M3 | L-5 | non-exp | 0.0034 | n/a |
| *51* | M3 | L-5 | non-exp | 0.0054 | n/a |
| *52* | M3 | L-5 | non-exp | 0.0075 | n/a |
| *53* | M3 | L-3 | non-exp | 0.0271 | 1.476855 |
| *54* | M3 | L-3 | non-exp | 0.0214 | 1.37734 |
| *55* | M3 | L-3 | non-exp | 0.032 | 1.488795 |
| *56* | M3 | L-3 | non-exp | 0.0184 | 1.302179 |
| *57* | M3 | L-3 | non-exp | 0.0117 | 1.109179 |
| *58* | M3 | L-3 | non-exp | 0.0191 | 1.316902 |
| *59* | M3 | L-1 | non-exp | 0.0318 | 1.482111 |
| *60* | M3 | L-1 | non-exp | 0.0306 | 1.504256 |
| *61* | M3 | L-1 | non-exp | 0.0529 | 1.794393 |
| *62* | M3 | L-1 | non-exp | 0.055 | 1.821151 |
| *63* | M3 | L-1 | non-exp | 0.0489 | 1.803298 |
| *64* | M3 | L-1 | non-exp | 0.0485 | 1.669553 |
| *65* | M3 | L-0 | non-exp | 0.066 | 3.59388 |
| *66* | M3 | L-0 | non-exp | 0.0558 | 3.562817 |
| *67* | M3 | L-0 | non-exp | 0.0417 | 3.539815 |
| *68* | M3 | L-0 | non-exp | 0.0621 | 3.653829 |
| *69* | M3 | L-0 | non-exp | 0.0442 | 3.525968 |
| *70* | M3 | L-0 | non-exp | 0.0406 | 3.55831 |
| *71* | M4 | L-5 | non-exp | 0.0034 | n/a |
| *72* | M4 | L-5 | non-exp | 0.005 | n/a |
| *73* | M4 | L-5 | non-exp | 0.0193 | n/a |
| *74* | M4 | L-5 | non-exp | 0.007 | n/a |
| *75* | M4 | L-5 | non-exp | 0.0064 | n/a |
| *76* | M4 | L-3 | non-exp | 0.0204 | 1.39 |
| *77* | M4 | L-3 | non-exp | 0.0149 | 1.19 |
| *78* | M4 | L-3 | non-exp | 0.0078 | 1.03 |
| *79* | M4 | L-3 | non-exp | 0.0203 | 1.30 |
| *80* | M4 | L-3 | non-exp | 0.0156 | 1.20 |
| *81* | M4 | L-3 | non-exp | 0.0145 | 1.26 |
| *82* | M4 | L-1 | non-exp | 0.0348 | 1.61 |
| *83* | M4 | L-1 | non-exp | 0.0476 | 1.70 |
| *84* | M4 | L-1 | non-exp | 0.0602 | 1.85 |
| *85* | M4 | L-0 | non-exp | 0.0403 | 3.45 |
| *86* | M4 | L-0 | non-exp | 0.0395 | 3.54 |
| *87* | M4 | L-0 | non-exp | 0.0402 | 3.64 |
| *88* | M4 | L-0 | non-exp | 0.0421 | 3.40 |
| *89* | M4 | L-0 | non-exp | 0.0399 | 3.32 |
| *90* | M4 | L-0 | non-exp | 0.0445 | 3.88 |
| *91* | M4 | L-0 | non-exp | 0.0508 | 3.66 |
| *92* | M4 | L-0 | non-exp | 0.0703 | 3.73 |
| *93* | M4 | L-0 | non-exp | 0.0373 | 3.46 |
| *94* | M4 | L-5 | exp | 0.0016 | n/a |
| *95* | M4 | L-5 | exp | 0.0013 | n/a |
| *96* | M3 | L-5 | exp | 0.0016 | n/a |
| *97* | M3 | L-5 | exp | 0.0031 | n/a |
| *98* | M3 | L-5 | exp | 0.0058 | n/a |
| *99* | M4 | L-5 | exp | 0.0007 | n/a |
| *100* | M4 | L-3 | exp | 0.0227 | 1.38 |
| *101* | M3 | L-3 | exp | 0.0449 | 1.644028 |
| *102* | M3 | L-3 | exp | 0.0269 | 1.477131 |
| *103* | M3 | L-3 | exp | 0.0473 | 2.023534 |
| *104* | M4 | L-3 | exp | 0.0368 | 1.09 |
| *105* | M4 | L-3 | exp | 0.0211 | 1.26 |
| *106* | M3 | L-1 | exp | 0.0534 | 1.847112 |
| *107* | M3 | L-1 | exp | 0.0518 | 1.787018 |
| *108* | M4 | L-1 | exp | 0.0494 | 2.05 |
| *109* | M3 | L-0 | exp | 0.0416 | 3.432618 |
| *110* | M4 | L-0 | exp | 0.0426 | 3.61 |
| *111* | M4 | L-0 | exp | 0.0423 | 3.37 |
| *112* | M4 | L-0 | exp | 0.0439 | 2.824387 |
| *113* | M3 | L-0 | exp | 0.04 | 3.655183 |
| *114* | M4 | L-0 | exp | 0.0425 | 3.51 |


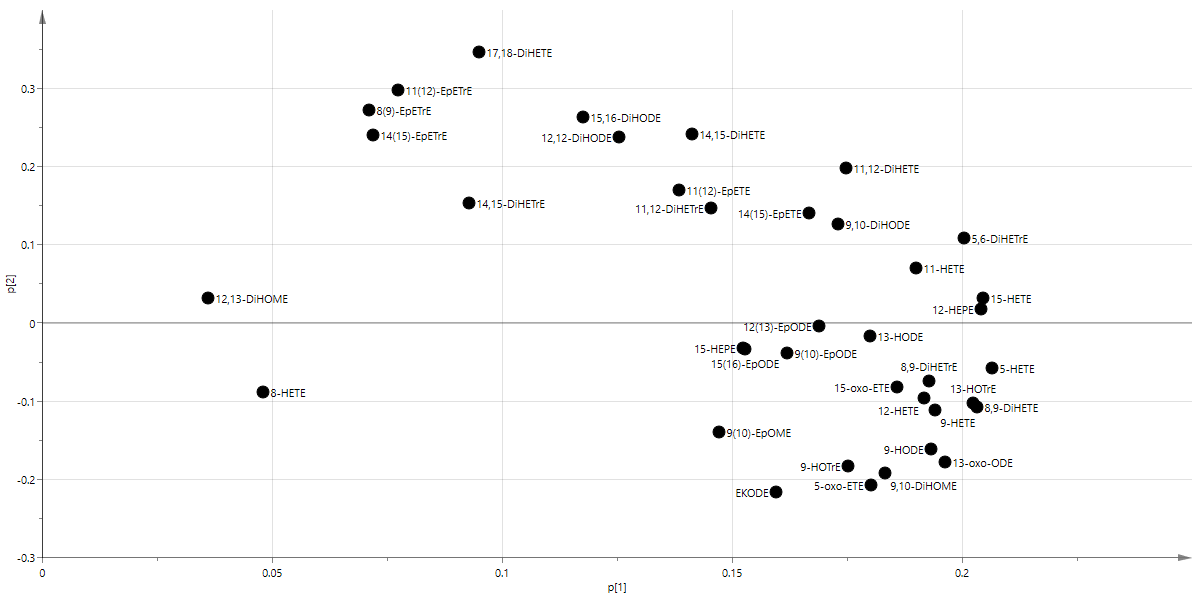


Figure S1. PCA loading plot showing the contribution of each oxylipin to the positions of the damselfly larvae and adult specimens.

*Figure S2 a). Coefficients of variation (%CV) for oxylipins at instar L-5. Oxylipins responsive to effluent exposure are marked in black.*


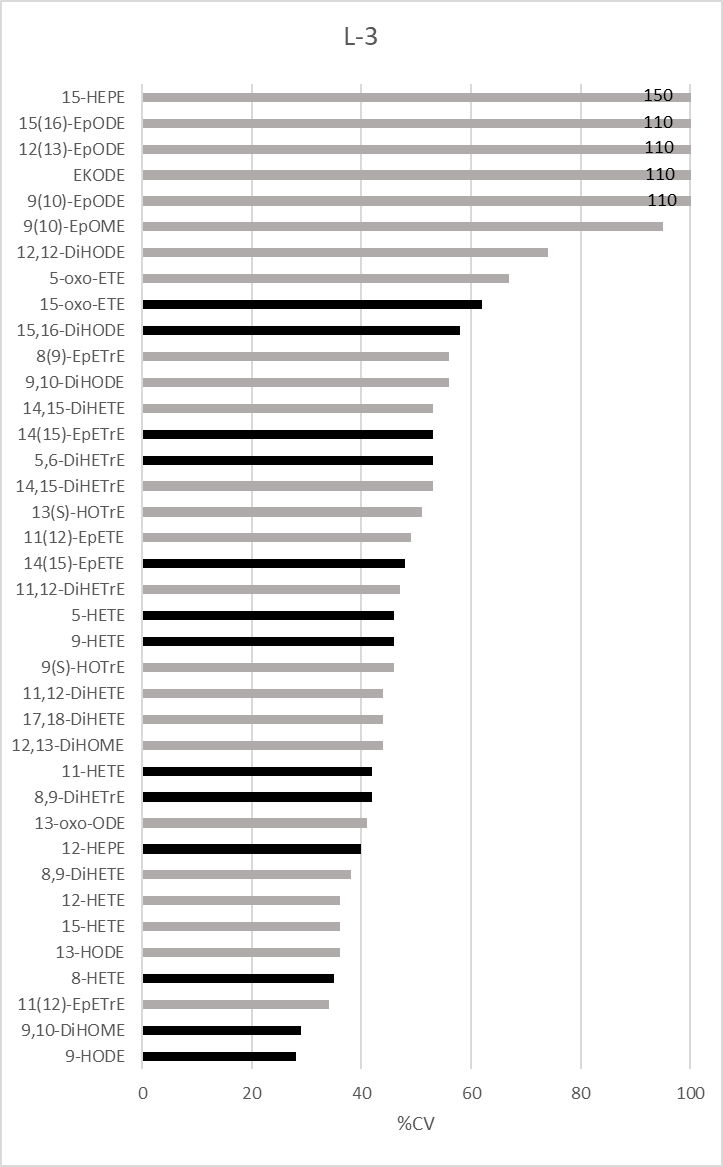


*Figure S2 b). Coefficients of variation (%CV) for oxylipins at instar L-3. Oxylipins responsive to effluent exposure are marked in black.*

*Figure S2 c). Coefficients of variation (%CV) for oxylipins at instar L-1. Oxylipins responsive to effluent exposure are marked in black.*

Figure S2 d). Coefficients of variation (%CV) for oxylipins at instar L-0. Oxylipins responsive to effluent exposure are marked in black.

**Description of Umeå wastewater treatment plant**

Umeå wastewater treatment plant serves approximately 100000 inhabitants and has a capacity corresponding to 166 000 population equivalent (Vakin, 2018). It covers rural areas, Umeå city, semi-industrial areas and a university hospital. The wastewater flow is 30000 m3/day and it is subjected to the following treatments: mechanical, chemical (flocculation with FeCl3), biological (active sludge), and chemical (flocculation with FeCl3). Treatments that remove nitrogen or disinfecting the wastewater are not used. The removal efficiency of phosphorous and organic material is above 95%. Details of effluent characteristics can be found in the environmental report. Reference: Vakin, Miljörapport Öns avloppsreningsverk år 2017, Umeå 2018

**Extraction protocol comparison**

Deuterated internal standard recoveries were determined for four extraction protocols commonly used for oxylipin analysis and/or metabolite analysis of invertebrates: (i) a solid liquid extraction (SLE) protocol previously used for oxylipin analysis in damselfly larvae (Späth et al., 2020), (ii) a standard SLE protocol used for metabolomics analysis of various matrices (Diamanti et al., 2019), (iii) a micro Quick, Easy, Cheap, Effective, Rugged, and Safe (QuEChERS) method used for non-targeted analysis of invertebrates (Berlioz-Barbier et al., 2018), and (iv) an ultrasonic extraction (USE) protocol used for invertebrates (Huerta et al., 2015). In addition, each examined protocol was carried out followed by a solid phase extraction (SPE) step, as used for oxylipin analysis in plasma (Gouveia-Figueira et al., 2015).

Damselfly larvae (22 mg ± 2 wet weight) were spiked in triplicate with internal standards (medium level, see Table S4) and extracted. Peak areas for each internal standard were normalized against the recovery standard and compared to ratios in methanol. Overall, the two SLE methods resulted in the greatest recoveries for the majority of internal standards (Figure S3). With the tested material (Oasis HLB, 60 mg, Waters, Milford, MA, USA), SPE did not improve recoveries for any of the tested methods and was therefore omitted. Small adjustments in terms of solvents and volumes resulted in the final optimized method described in the Materials and Methods section of the article.


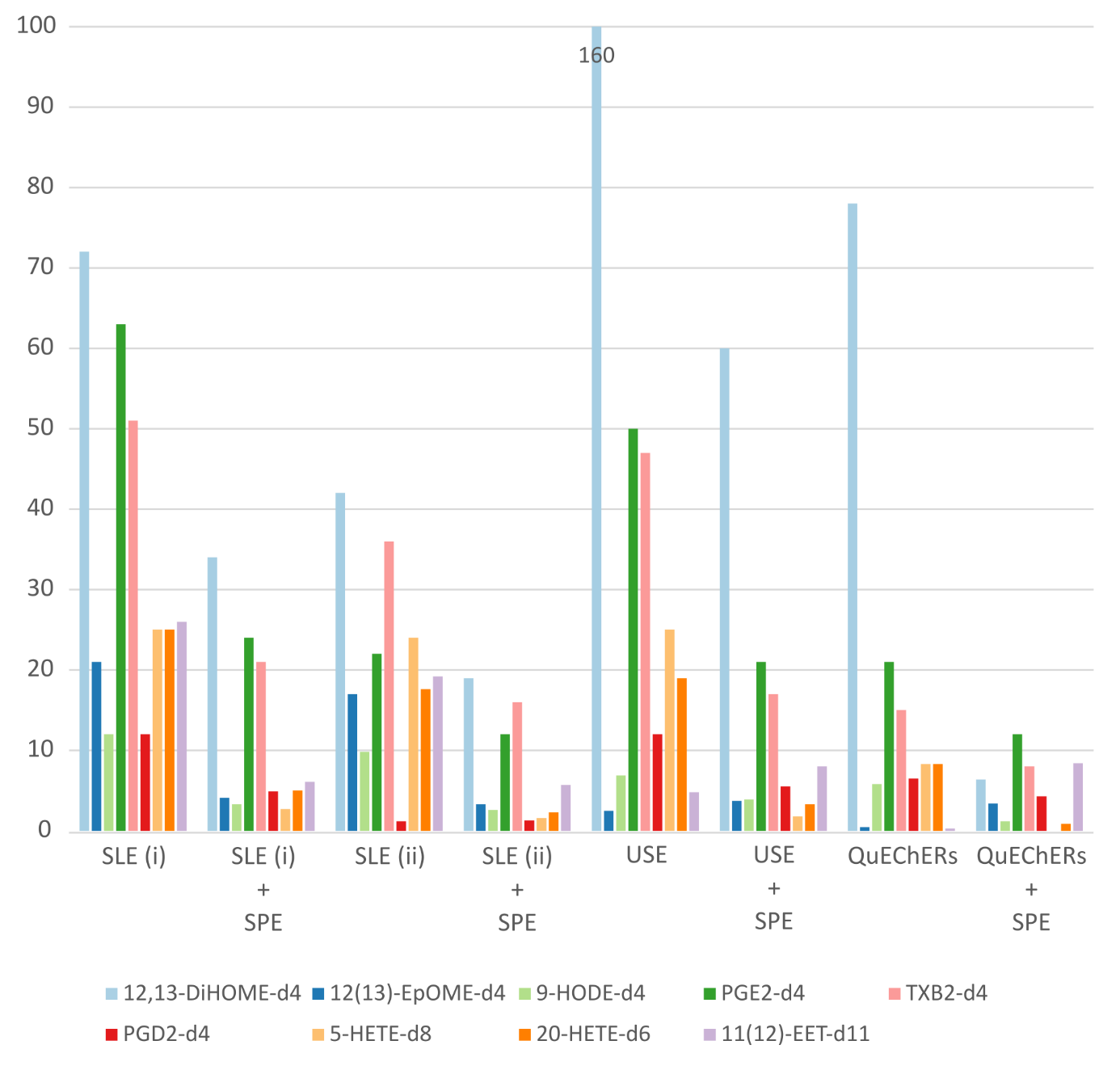


Figure S3. Deuterated internal standard recoveries for four extraction methods with and without an additional SPE step.

Berlioz-Barbier, A., Buleté, A., Fildier, A., Garric, J., Vulliet, E., 2018. Non-targeted investigation of benthic invertebrates (Chironomus riparius) exposed to wastewater treatment plant effluents using nanoliquid chromatography coupled to high-resolution mass spectrometry. *Chemosphere* 196, 347–353. https://doi.org/10.1016/j.chemosphere.2018.01.001

Diamanti, K., Cavalli, M., Pan, G., Pereira, M.J., Kumar, C., Skrtic, S., Grabherr, M., Risérus, U., Eriksson, J.W., Komorowski, J., Wadelius, C., 2019. Intra- and inter-individual metabolic profiling highlights carnitine and lysophosphatidylcholine pathways as key molecular defects in type 2 diabetes. *Sci Rep* 9, 1–13. https://doi.org/10.1038/s41598-019-45906-5

Gouveia-Figueira, S., Späth, J., Zivkovic, A.M., Nording, M.L., 2015. Profiling the oxylipin and endocannabinoid metabolome by UPLC-ESI-MS/MS in human plasma to monitor postprandial inflammation. *PLOS ONE* 10, e0132042. https://doi.org/10.1371/journal.pone.0132042

Huerta, B., Jakimska, A., Llorca, M., Ruhí, A., Margoutidis, G., Acuña, V., Sabater, S., Rodriguez-Mozaz, S., Barcelò, D., 2015. Development of an extraction and purification method for the determination of multi-class pharmaceuticals and endocrine disruptors in freshwater invertebrates. *Talanta* 132, 373–381. https://doi.org/10.1016/j.talanta.2014.09.017

Späth, J., Nording, M., Lindberg, R., Brodin, T., Jansson, S., Yang, J., Wan, D., Hammock, B., Fick, J., 2020. Novel metabolomic method to assess the effect-based removal efficiency of advanced wastewater treatment techniques. *Environ. Chem.* https://doi.org/10.1071/EN19270
